# Accuracy of Measurements of Thermophysical Parameters by Dual-Beam Thermal-Lens Spectrometry

**DOI:** 10.3390/nano13030430

**Published:** 2023-01-20

**Authors:** Vladislav R. Khabibullin, Mladen Franko, Mikhail A. Proskurnin

**Affiliations:** 1 Analytical Chemistry Division, Chemistry Department, M.V. Lomonosov Moscow State University, d. 1, str. 3, Lenin Hills, GSP-1 V-234, Moscow 119991, Russia; 2 Laboratory for Environmental and Life Sciences, University of Nova Gorica, Vipavska 13, Rožna Dolina, 5000 Nova Gorica, Slovenia

**Keywords:** thermal-lens spectrometry, accuracy and trueness, mode-mismatched schematic, measurements of thermal diffusivity

## Abstract

Thermal-lens spectrometry is a sensitive technique for determination of physicochemical properties and thermophysical parameters of various materials including heterogeneous systems and nanoparticles. In this paper, we consider the issues of the correctness (trueness) of measurements of the characteristic time of the thermal-lens effect and, thus, of the thermal diffusivity determined by dual-beam mode-mismatching thermal lensing. As sources of systematic errors, major factors—radiation sources, sample-cell and detector parameters, and general measurement parameters—are considered using several configurations of the thermal-lens setups, and their contributions are quantified or estimated. Furthermore, with aqueous ferroin and Sudan I in ethanol as inert colorants, the effects of the intermolecular distance of the absorbing substance on the correctness of finding the thermophysical parameters are considered. The recommendations for checking the operation of the thermal-lens setup to ensure the maximum accuracy are given. The results obtained help reducing the impact of each investigated factor on the value of systematic error and correctly measure the thermophysical parameters using thermal-lens spectrometry.

## 1. Introduction

Photothermal spectroscopy (PTS) solves a broad range of problems in the analysis and characterization of complex and heterogeneous systems based on the assessment of their thermal and optical properties [1,2,3]. Thermal-lens spectrometry (TLS) occupies an essential position in the group of photothermal methods and is used to solve many physicochemical problems [3,4,5,6]. TLS is used for monitoring the course of chemical reactions [7,8] and is widespread in biochemical analysis [4]. Due to the high sensitivity of TLS and fast response time, the method has proven itself as a detector in high-performance liquid chromatography (HPLC) [9,10,11], enzyme immunoassays [12], microfluidics [13], and capillary electrophoresis [14,15], as well as in various sorption/extraction separation methods [16,17]. The method is used in finding the luminescence quantum yields [18,19]. A broad selection of lasers allows TLS to solve research problems using UV/vis [20] and IR ranges [21,22,23]. The high sensitivity of TLS to the sample composition made it possible to study the effect of electrolytes and organic solvents on the polarizability of water molecules [24,25]. In comparison with transmission photometry, TLS makes it possible to more accurately determine the stability constants of complexes [26].

Currently, most TLS applications rely on the use of the dual-beam (pump–probe) configuration [3,4,5,27]. As a rule, a probe beam is a continuous He–Ne laser. For the excitation source, gas (usually, argon ion) [28,29,30,31], diode [32,33], and solid-state (Ti:Sapphire) [7,27,34] lasers operating in the UV, visible, and IR ranges are used. Both pulsed [35], and cw sources [36,37] are applied. The selection of the excitation laser is determined by the research task. Dual-beam TLS also differs in the direction of laser beam propagation through the sample. Three options are possible: (i) collinear (the beams are strictly co-axial) [31,38], (ii) the beams intersect in the sample at a small angle (usually 1.5°) [34,39], and (iii) the crossed-beams configuration (an angle of 90°) [15,40]. The latter case is relatively rarely used in thermophysical studies due to the difficulty of aligning the beams relative to each other and the small sample volume. In this paper, we consider a two-beam collinear configuration only.

Apart the thermophysical and physicochemical studies themselves, in chemical analysis, thermophysical properties (thermal diffusivity, thermal conductivity, and heat capacity) of materials are more significant than optical parameters since they allow for a more selective identification by revealing the dynamics of changes in the composition and structure. Common methods for measuring thermophysical parameters (heat flow, non-stationary hot wire, protected hot plate, 3ω-method, etc.) [41] have some limitations (thermal convection, long time of measurement, and low sensitivity to chemical composition) and do not always allow online or real-time process monitoring. On the contrary, TLS is highly sensitive to rather slight changes in the sample composition and also requires minimal sample preparation [42,43]. Emphasis of TLS application in this area is placed on the accurate calculation of thermophysical properties of nanofluids [36,43,44,45,46], biodiesel [47,48] solid multicomponent systems [49], nanoparticles [50,51,52], quantum dots [53], glasses [54,55,56], and semiconductors [57].

Advantages of TLS are limited in case of substances with low absorbances (mainly for homogeneous systems) and low concentrations (mainly for heterogeneous systems). In the practice of thermal lens measurements, the calculation of thermophysical properties of an unknown sample is made in relation to a reference material. Commonly, water or organic solvents with accurately known parameters (ethanol, acetonitrile, chloroform, toluene, etc.) serve as reference materials [28,29]. At the same time, the main attention is paid to the sensitivity of the method [58], the decrease in determined analyte concentrations [59,60], reduction in the analysis time, and increase in sample throughput [61,62].

According to ISO 5725, the accuracy of a measurement method is regulated by two concepts: trueness and precision. Trueness is defined as the closeness of the arithmetic mean of a large number of test results and the true (or reference) value and describes the systematic error. Precision describes the random error and is defined as the closeness of measurement values to each other. Thus, the accuracy of the measurement is achieved as the combination of systematic and random errors.

In the practice of thermal-lens measurements, the issues of trueness are given less attention, and the available results are non-systemic [63,64]. In applied research, the main emphasis is on the precision of the results, improperly considering this as the only factor that governs the accuracy [50,53,65]. Some aspects of the trueness of thermal-lens measurements are considered in the context of optimizing methods for a particular analyte (and, as a rule, only in relation to the signal amplitude), within which an original setup is designed, and its operation parameters are optimized [66,67]. For instance, using an example of quantifying permanganate ions in water, the geometry of a dual-beam spectrometer with a mode mismatch was optimized [68]. Within the framework of the problem, the impact from the probe-beam waist position, confocal distance, and detector position on the signal was studied [68]. Independent studies on the influence of the excitation source (a Gaussian mode or a cylindrical incoherent light source) [64,69] and beam divergence on the thermal-lens signal are given; noise sources (light sources, sample, detector) are analyzed; the optimal ratio of the radius of the aperture pinhole to the beam radius for the maximum signal-to-noise ratio is proposed [64]. However, the values of systematic errors that arise during the adjustment and tuning of the optical components of the spectrometer and, most importantly, the quantitative effect of these errors on the measurement results are not stated in sufficient detail.

In this study, the factors that most affect the trueness of measurement of thermophysical parameters in dual-beam TLS are found and quantitatively described. Namely, they are fluctuations in the transverse spatial mode of excitation radiation, error in the size of beams of both lasers in the sample cell, cell positioning errors, the positioning of the probe laser at the detector, errors associated with the sample (absorbance and microimpurities), and errors related to the measurement parameters (mode-mismatching, the modulator frequency, the time of steady state, and the averaging of the transient curves). To compare the impact of these factors, several of the most common variants of dual-beam configurations in modulated continuous-wave operation using TEM_00_ mode lasers are considered.

## 2. Materials and Method

### 2.1. Thermal Lensing

All designations and constants that are found in the text are summarized in the nomenclature table, which is located in the Supplementary Materials, Table S1.

In TLS, the refractive index gradient that arises when a sample is irradiated by an excitation beam acts as a lens-like element called the thermal lens [3,4,5,6]. If the sample has a negative refractive index temperature coefficient (dn/dT), then the thermal lens expands the transmitted beam. The dual-beam TLS uses an excitation (pump) source, which is a high-power laser that is used to generate a thermal lens, and a probe, a low-power laser, by the change of which the thermal lens is recorded and analyzed. Shen et al. presented a robust and commonly used model [70] of a highly sensitive dual-beam mode-mismatched thermal-lens spectrometer configuration, in which the excitation beam waist was at the center of the sample and the probe beam waist was at a short distance ($z_{1}$) after the sample. The model was developed for beams with a Gaussian intensity distribution transverse mode (TEM_00_). To obtain the highest signal, the radii of the probe ($\omega_{p1}$) and excitation ($\omega_{e0}$) beams in the sample should not be equal (so called *mode mismatching*). Shen et al. also derived an equation for changing intensity on the axis of the probe beam with time $I\left(t\right)$ recorded by a detector located at a distance $z_{2}$ from the sample, Equation (1) below. In this case, the distance between the sample and the detector must be much greater than the confocal distance ($z_{2}$>>$z_{cp}$). The on-axis intensity of the probe beam mainly depends on: (a) the probe beam intensity at time *t* = 0, $I\left(0\right)$, (b) the mode-mismatch parameter *m*, indicating the ratio of the probe and excitation beam radii in the sample, (c) the distance parameter *V*, depending on the configuration, (d) the thermal-lens characteristic time $t_{c}$, and (e) *θ*, the thermooptical signal.

(1) $$I\left(t\right)=I\left(0\right){\left[1-\frac{\theta }{2}\tan^{-1}\left(\frac{2mV}{\left[{\left(1+2m\right)}^{2}+V^{2}\right]\left(t_{c}/2t\right)+1+2m+V^{2}}\right)\right]}^{2},$$

where the parameters *m*, *V*, and $t_{c}$ are defined as

(2) $$m={\left(\omega_{p1}/\omega_{e0}\right)}^{2},$$

(3) $$V=\frac{Z_{1}}{Z_{cp}}+\frac{Z_{cp}}{Z_{2}}\left[1+{\left(\frac{Z_{1}}{Z_{cp}}\right)}^{2}\right],$$

where $z_{cp}$ is the Rayleigh length ($z_{cp}=\pi \omega_{p0}^{2}/\lambda_{p}$) for the probe beam (mm); $\omega_{p0}$ is the probe beam waist radius (µm),

(4) $$t_{c}=\omega_{e0}^{2}/4D,$$

and

(5) $$\theta =\frac{P\alpha l}{k\lambda_{p}}\left(-\frac{dn}{dT}\right).$$

Here, *α* is the linear absorption coefficient of the sample; *l* is the cell path length (cm); *P* is the excitation laser power (W); $\lambda_{p}$ and $\lambda_{e}$ are the wavelengths of the probe and excitation lasers (nm), respectively; *k* is the thermal conductivity of the sample (W/(m·K)); and dn/dT is the temperature coefficient of the refractive index (the thermooptical constant).

Primary experimental data are transient (time-resolved) curves $I\left(t\right)$ fitted using Equation (1). The parameters $t_{c}\;$ and *θ*, from which the thermal diffusivity is calculated, are found from Equations (4) and (5) using the external measurements or theoretical values.

### 2.2. Spectrometers

**Figure 1:**
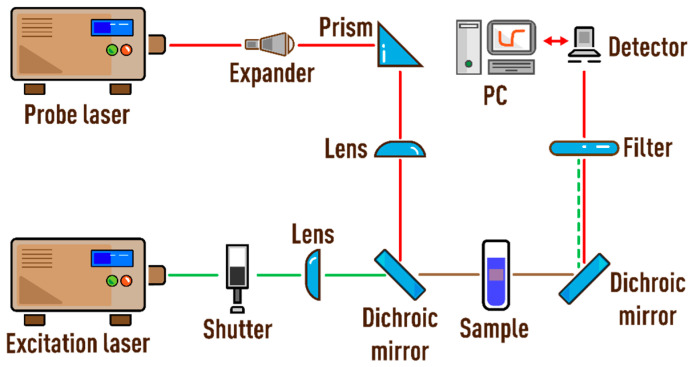
Schematic of the narrow-focused configuration of the thermal-lens spectrometer.

**Figure 2:**
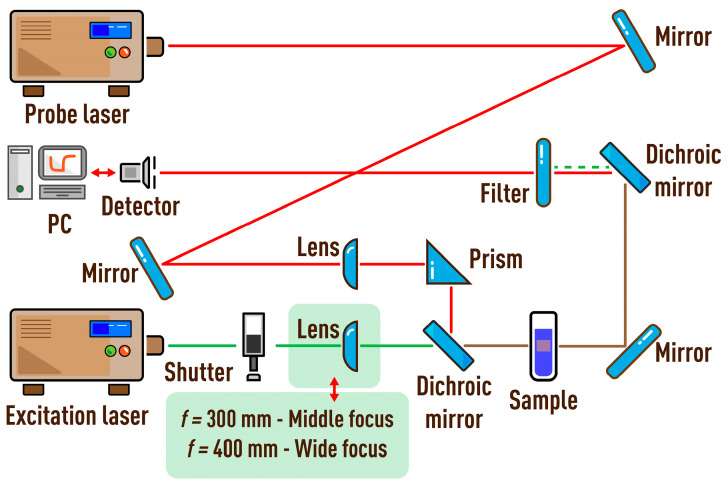
Schematics of middle-focused and wide-focused configurations of the thermal-lens spectrometer.

The characteristic times and thermal diffusivities were experimentally measured on a dual-beam spectrometer in several configurations. Schematic of a dual-beam thermal-lens spectrometer is shown in Figure 1 and Figure 2. A thermal lens is generated in a quartz cell (*l* = 10.00 mm) by radiation from an MGL-FN-532 solid-state laser (Changchun New Industries Optoelectronics Tech. Co., Ltd., Changchun, China; TEM_00_ mode) with a wavelength of 532.0 nm (TEM_00_). The probe laser was an HNL050L He–Ne laser (ThorLabs, Newton, NJ, USA) with a wavelength of 632.8 nm (TEM_00_, 5.0 mW). The parameters of lasers are presented in Table 1 and Table 2. 

The radiation of the excitation laser passes through a modulator (shutter). The shutter (model SH05, ThorLabs, Newton, NJ, USA) is controlled via an analog-to-digital and digital-to-analog converter board (ADC–DAC), model c8051Fx-DK (Silicon Labs, Boston, MA, USA) connected to a personal computer. The recording of the thermal-lens signal starts at the moment the shutter is opened, synchronously. After closing the shutter, the signal from the photodiode continues to be recorded for the same period. At the moment of the next opening of the shutter, the recording of the signal stops, and a new cycle begins. The personal computer receives data from the ADC to the homemade software (C++ programming, Borland Corp., Austin, TX, USA), in which all measurement cycles are formed, displayed, and stored in the form of transient curves (signal intensity vs. time) [71]. To check the power of lasers, an Optronics Nova II power meter (Ophir Optronics Solutions, Jerusalem, Israel) with a highly sensitive 3A thermoelectric sensor was used. The operating parameters of the measurements are summed up in Table 3. The transient curves were averaged over 10 min.

### 2.3. Measurement Procedures

Provided the geometrical parameters of the configuration (*V* and *m*), the power of the excitation laser, and the thermophysical and optical parameters of the object are known, from the shape of the transient curve, the characteristic time can be found. Since the parameters *m* and *V* are thermal-lens setup constants, Equation (1) can be represented in the following form:

(6) $$I\left(t\right)=I\left(0\right){\left[1-0.5{\theta \tan}^{-1}\left(a/\left(b\left(t_{c}/2t\right)+c\right)\right)\right]}^{2},$$

where *a*, *b*, and *c* are constants: *а* = 2*mV*, *b* = (1 + 2*m*)^2^ + *V*^2^, and *c* = 1 + 2*m* + *V*^2^. The theoretical transient curve in the work was calculated using Equation (1), where the parameters *θ* and $t_{c}$ were found from the reference thermophysical data and absorbance measurements.

To find the characteristic time and thermal diffusivity, we used the full development of the thermal field and the transition of the transient curve to a stationary state. This means that instead of $I\left(t\right)$, we use $I\left(\infty \right)$, the intensity of the probe beam when the stationary (steady) state of the thermal lens is reached.

Considering the above, the characteristic time can be derived from Equation (1) by representing it as a function of time:

(7) $$\;\tilde{t_{c}}\left(t\right)=\left[\left(a/\tan\left[2\cdot \frac{1-\sqrt{I\left(t\right)/I\left(\infty \right)}}{\vartheta }\right]\right)-c\right]\cdot \frac{2t}{b},$$

where $I\left(\infty \right)$ is the intensity of the probe beam when the stationary state of the thermal lens is reached

(8) $$\vartheta =2\left[1-\frac{I\left(\infty \right)}{I\left(0\right)}\right]/\tan^{-1}\left(a/c\right)\;.$$

Thus, at each moment *t* of the development of the time-resolved curve $I\left(t\right)$, we obtain the effective characteristic time. Thermal diffusivity ($\tilde{D}\left(t\right)$, referred to as effective thermal diffusivity) is calculated for each value $\tilde{t_{c}}\left(t\right)\;$ at time *t*. For the transition from effective $t_{c}$ and *D* to true values, the average value from the first 100 ms of the functions $\tilde{t_{c}}\left(t\right)\;$ and $\tilde{D}\left(t\right)$ was found. 

Additionally, the transient curves were normalized: either to the largest value of $I\left(t\right)/I\left(0\right)$ or normalized to the range of 0 ÷ 1. In the latter case, the following equation was applied:

(9) $$\tilde{I}\left(t\right)=\left[I\left(t\right)-I\left(\infty \right)\right]/\left[I\left(0\right)-I\left(\infty \right)\right].$$

The sizes of the laser beams were measured by the moving blade method described in detail in [72,73,74]. The measurements were conducted before each experiment 5–7 times at intervals of 5–10 min; for this, a digital micrometer (MKTs type, LLC NPP CHIZ, Chelyabinsk, Russia, corresponding to the GOST 6507-90 State Standard of Russian Federation) was used with an absolute error of ±2 μm, and a Nova 2 power meter (Ophir Optronics Solutions, Jerusalem, Israel) was used. All optical and mechanical components were acquired from Thorlabs (Newton, NJ, USA), except for the excitation laser, which was acquired from CNI (Changchun, China). The parameters of the lasers are presented in Table 1 and Table 2. To focus the beams, biconvex lenses with focal lengths of 20.0, 30.0, and 40.0 cm were used.

#### 2.3.1. Auxiliary Measurements

An Agilent Cary 4000 spectrophotometer (Agilent, Mulgrave, Australia) was used for recording of UV/Vis spectra, *l* = 1–10 mm, cell volume 0.3–3 cm^3^. The pH values were measured by an inoLab pH Level 1 pH-meter (WHW, Weilheim, Germany) with a glass pH-selective electrode (precision ±5%). Solutions were mixed with a Biosan MMS 3000 automatic mixer (Riga, Latvia).

### 2.4. Reagents

All reagents and materials were purchased from Merck (Darmstadt, Germany) and were of cp grade. Pure organic solvents were used as model solutions: acetonitrile, toluene, ethanol, and chloroform. Solutions of Sudan I in ethanol with a concentration of 0.1 μmol/L and an aqueous solution of ferroin (tris(1,10-phenanthrolinate) iron(II) sulfate) with a concentration of 2.5 μmol/L were used as reference samples. To study the bias caused by the concentration of solutions, aqueous solutions of ferroin were prepared in the range of 1.0–50.0 μmol/L. All samples were prepared by diluting a 25 mmol/L ferroin stock solution with deionized water.

## 3. Results and Discussion

**Figure 3:**
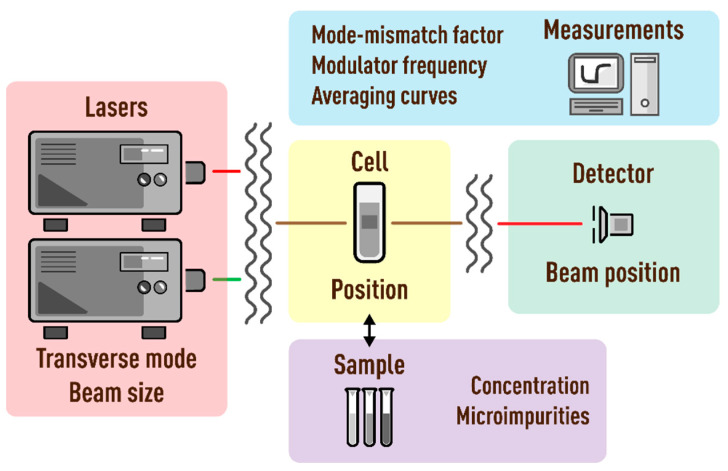
Main groups of factors affecting the systematic error in determination of thermophysical parameters by thermal-lens spectrometry.

The paper considers and quantifies the factors affecting only the value of systematic error of measurements of the characteristic time and thermal diffusivity by the method of dual-beam TLS. Figure 3 is a chart of the main sources of errors considered in the study that affect the value of systematic error of $t_{c}$ and *D*. Conventionally, they can be divided into five groups. They include lasers (or radiation sources), cell, detector, sample, and measurement parameters. The paper is structured in such a way that each section or factor influencing the result of measuring thermophysical parameters is independent and can be considered separately from the others. In this case, the presentation of each group of parameters continues sequentially according to the principal scheme of the thermal-lens spectrometer (Figure 1 and Figure 2), starting with lasers and ending with questions regarding the measurement parameters on the example of specific objects. In conclusion, a general error scheme is given with an assessment of the influence of each group of factors on the trueness of determined thermophysical parameters.

The influence of factors is shown by the example of the operation of a dual-beam far-field thermal-lens spectrometer. The choice of operating parameters of the built setups is based on an analysis of the literature over the past decades and, in general, considers the main parameters used for dual-beam thermal-lens spectrometry (examples of mainly used thermal-lens spectrometers are given in the Supplementary Materials, Table S2). These are most commonly used configurations in thermal-lens measurements in applied chemistry and thermophysical measurements [3,4,5,6,61,65]. The spectrometers are built in three configurations (Figure 1 and Figure 2). The spatial scale of beams and beam ratios is common for all configurations and previously optimized for light-absorption and thermophysical measurements. Additionally, the selection of this beam size scale mostly reduces the vibration effect of the optical components of the spectrometer, which is not considered here. 

However, to consider the differences in thermal-lens measurements for various practical tasks, the spectrometers show different conditions within this common scale. The main parameter and, at the same time, the main difference between the built configurations is the waist size of the excitation beam *ω_e_*_0_. Within the framework of the selected spatial scale, we used the values of $\omega_{e0}$: 33, 42, and 82 µm (Table 3). Thus, for other averaged values of the geometrical parameters, we obtained a difference in the waist of the excitation laser beam by more than a factor of two, which significantly affects the experimental results. As the characteristic time according to Equation (4) depends on *ω_e_*_0_, when the waist changes, the experimental value of $t_{c}$ changes. This leads to a change in the accuracy of the experimental value of thermal diffusivity, which is the key property of consideration of this study. Thus, in this work, the values of the characteristic time obtained from the experimental transient curve and calculated theoretically by Equation (4) were used as a criterion for the influence of a factor on the trueness of measurements. The ratio of the parameters of an experimental transient curve and the theoretical curve is used as a criterion of trueness. It should be noted that this study does not compare and optimize the spectrometer configurations, which is considered elsewhere [3,14,71,75,76,77]; only the different conditions due to different beam-waist sizes are considered.

For ease of discussion, each configuration is given a name in accordance with the waist size. From this point on, the configuration with $\omega_{e0}$ = 33 µm is referred to as ‘*narrow-focused*’; 42 µm, ‘*middle-focused*’; and ‘*wide-focused*’ where the waist was 82 µm.

To classify the impact of factors on the correctness of finding $t_{c}$ and *D*, based on the magnitude of the value of systematic error that each factor introduces, we introduced the following concepts. The bias in finding $t_{c}$ and *D* less than 1% is ‘*insignificant*’; 1–5%, ‘*moderate*’; 5–10%, ‘*high*’; and more than 10%, ‘*unacceptable*’. The latter value is usually beyond the accuracy required for TLS assessment of thermal diffusivity (e.g., for nanofluids, it is comparable to the nanofluid effect itself). Furthermore, in the case of chemical analysis by thermophysical parameters, such an accuracy, it does not allow sample comparison and identification. As a whole, such error ranking helps us rating the varied factors according to their significance. To exclude errors associated with chemical transformations, we used true solutions with precisely known thermal and optical properties. Chemical reactions and specific disperse systems (micellar solutions or nanoparticle or quantum-dot dispersions, etc.), as well as factors related to the dispersity of solutions that affect thermophysical parameters, $t_{c}$ and *D*, are not considered here.

### 3.1. Excitation Radiation Source 

Systematic changes in the intensity and divergence of the probe laser beam introduce an insignificant value of systematic error of thermophysical parameters and will not be considered here. Changes in the divergence of the probe beam up to 10% lead to a bias of no more than 1% and is insignificant in relation to the determination of the characteristic time and thermal diffusivity.

Incorrect measurement of the probe-beam radius at the cell center is insignificant since it does not affect the shape of the curve and the signal. Thus, a bias in the radius of the probe beam of 20% leads to systematic errors of the characteristic time and thermal diffusivity of <1%. This manifests itself noticeably if the real radius changes by two or more times from the radius determined experimentally but is not considered here. The same is not true for the excitation laser, where changes in the transverse spatial mode have a strong influence on $t_{c}$ and *D*. Let us consider this factor in more detail.

#### 3.1.1. Periodic Changes of the Transverse Spatial Mode of Excitation Radiation

The stable operation of the excitation laser, most crucially beam divergence, determines the stability of the temperature gradient in the sample and, therefore, the accuracy of thermophysical parameters of the test sample. Changes in the beam divergence cause the radius of the waist and its position to shift, and this, in turn, leads to a change in the apparent characteristic time. Hence, the main requirement for the excitation beam is its stability of divergence and, to a lesser extent, of intensity.

The excitation intensity affects the amplitude thermal-lens signal only and does not affect the shape of the transient curve from which the thermophysical parameters are measured, Equation (1). Hence, the irradiation power does not introduce a bias into the measured $t_{c}\;$ and *D*. On the other hand, the beam divergence, which affects the radius of the waist and its position, has a significant effect on the calculation, and it will be considered in detail below.

Changes in beam radius about <1% are insignificant since they do not cause errors in determined values of the characteristic time and thermal diffusivity larger than 1%. To the contrary, changes in beam divergence of >1% contribute significantly to the systematic error. Let us consider long-term fluctuations (>10 min), which, due to their duration, may not be noticeable with a single measurement of the beam radius. This behavior of the excitation laser is revealed in long-term measurements of the beam waist. Often, this can be neglected accepting the fact of stable long-term operation of the instrument without more verification. Hence, if long-term measurements are needed, long-term variation in the excitation beam divergence can appear on the transient curves as a change in the apparent characteristic time. Therefore, it results in different values of determined thermal diffusivity of the test sample. Fluctuations in the excitation beam divergence, even in the range of 5%, have a serious effect on the trueness of $t_{c}\;$ and *D*.

**Figure 4:**
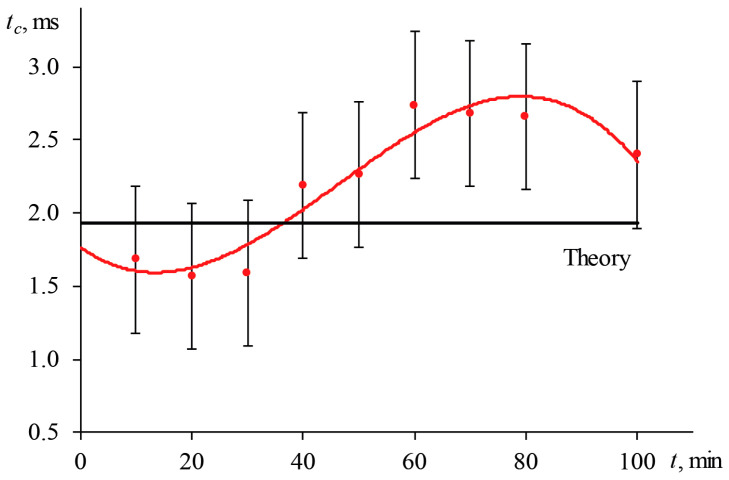
Change in the characteristic time of thermal-lens measurements in an aqueous solution of ferroin (2.5 μmol/L); narrow-focused configuration ($\omega_{e0}$ = 33 µm, Figure 1, Table 3).

**Figure 5:**
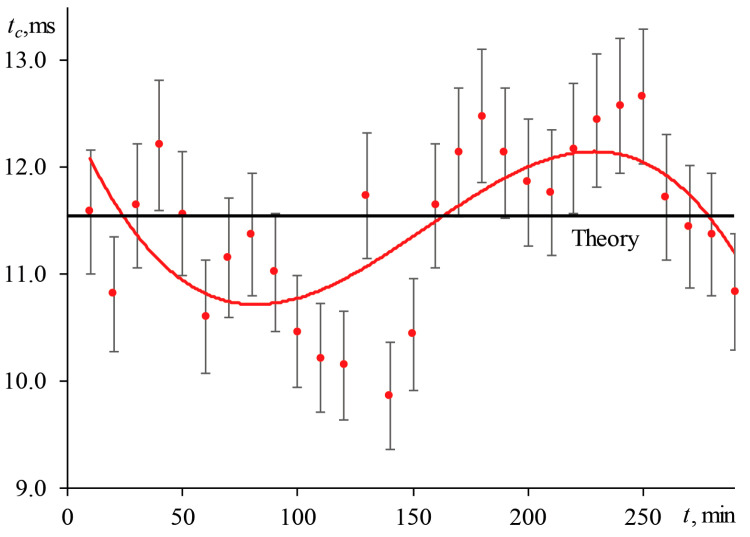
The behavior of the characteristic time of an aqueous solution of ferroin (2.5 μmol/L); wide-focused configuration ($\omega_{e0}$ = 82 µm, Figure 2, Table 3).

For the narrow-focused configuration (the scheme, Figure 1; the operating parameters, Table 3), the bias in characteristic time is ±0.6 ms (31%), which is commensurate with the value of $t_{c}$ itself (for a 2.5 μmol/L aqueous solution of ferroin, $t_{c,\;theor}$ = 1.93 ms). By conducting measurements under such conditions, it is impossible to obtain correct results; the values of the thermophysical parameters differ from the theoretical ones (Figure 4). Deviations from $t_{c,\;theor}$ in the specified period are unacceptable; they reach more than 40%, and thermal diffusivity can deviate by 30% from the true value. For the wide-focused configuration (Table 3), where for the same sample at $\omega_{e0}$ = 82.0 ± 1.0 µm, $t_{c,\;theor}$ = 11.55 ms (Figure 5) changes in the excitation beam radius led to a bias of 10% in $t_{c\;}$. 

**Figure 6:**
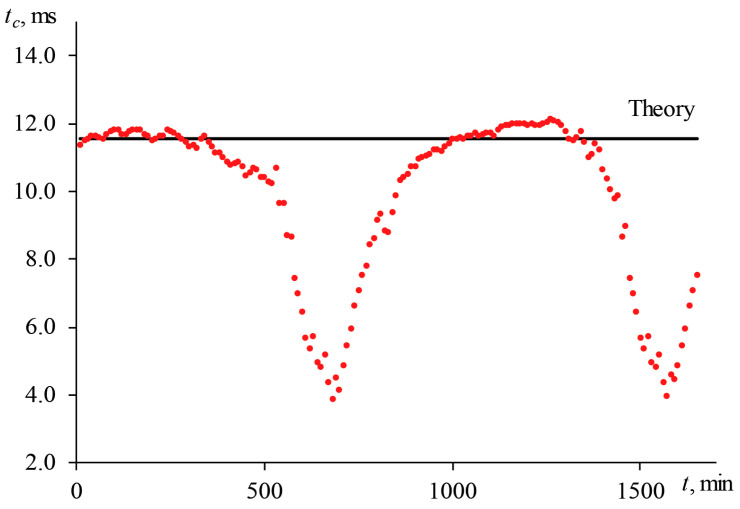
Behavior of the characteristic time of an aqueous solution of ferroin (2.5 μmol/L) with measurements on a wide-focused configuration ($\omega_{e0}$ = 82 µm, Figure 2, Table 3) for a long period.

In the presence of a longer drift with a period of more than 100–150 min, the analysis of the transient curves for the first 30–60 min of the experiment may not reveal deviations in the divergence of the excitation beam. In this case, the results obtained after 5–6 h may differ from the initial data. Figure 6 shows the behavior of the characteristic time over more than 24 h of measurements with the wide-focused configuration of the same sample.

For the first 350–400 min, $t_{c\;}$ fluctuates around the theoretical calculation from the measured diameter of the excitation beam in the cell before the start of measurements, which was 164 ± 2 µm. In this case, the previously described periodic changes in $t_{c}\;$ of 10% are observed. Subsequently, the apparent characteristic time decreases reaching a minimum value of 4.1 ± 0.4 ms after ca. 11 h (700 min). In this case, the measured diameter of the excitation beam was 104 ± 2 µm. Then the experimental $t_{c\;}$ again returns to its initial value and fluctuates around the theoretical value of 11.55 ms. In this case, the diameter of the beam also returns to the initial state (164 ± 2 µm) after 16 h. Later, changes in the beam divergence are repeated, as can be seen in Figure 6. Thus, long-term changes are found. 

Summarizing, one can conclude that, having a laser with a long-term systematic change in divergence of ca. 22.4% and short-term fluctuations of 10%, a deviation from true value in thermal diffusivity of more than 60% will be present, which is unacceptable for both chemical analysis and thermophysical (or technical) applications.

Thus, changes in the divergence of the excitation laser introduce a significant systematic error into the thermophysical parameters of the sample. To obtain correct results, either a stable excitation laser with changes in divergence of no more than 10% is required, or a beam radius measurement must be performed before each analysis (measurement repetition, at least once per 30 min); thermal-lens measurements should not take more than 2 h (at the same time, the averaging of transient curves should be carried out over all measurements performed within this time).

Another approach is measurements using the internal standard, relative to a reference sample with well-known thermophysical parameters (in such cases, analysis and averaging should not be conducted for more than 10 min). However, in this case, the confidence interval of the measurements increases, since the number of time-resolved curves for averaging decreases.

#### 3.1.2. Error in Beam Radius Measurement

**Figure 7:**
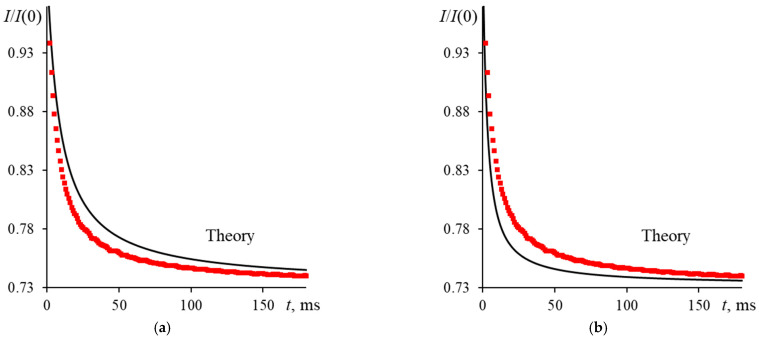
Normalized transient curves for an aqueous solution of ferroin (2.5 μmol/L) with incorrect measurement of the beam radius in the sample, where: (**a**) is the true radius of the excitation beam is less than the measured one; (**b**) is the true radius of the excitation beam is larger than the measured one; narrow-focused configuration ($\omega_{e0}$ = 33 µm, Figure 1, Table 3).

An incorrectly measured radius of the excitation beam ($\omega_{e0}$) in the cell leads to an incorrect thermophysical parameters. As stated above, changes in beam divergence affect the waist radius resulting in a change in the apparent characteristic time. Hence, a widespread problem is the absolute measurement error of the excitation beam radius. Let us consider this problem with an example of operation of a narrow-focused configuration (Figure 1, Table 3); the measurements were made for an aqueous solution of ferroin (2.5 μmol/L). If there is no measurement error in the beam radius in the cell, we will acquire the full ratio of the experimental and theoretical curves. Otherwise, we would see their inconsistency with each other. In this case, if the true radius of the beam is larger than the apparent one, a negative deviation is observed (Figure 7a), and the experimental curve lies lower than the theoretical one, and the resulting characteristic time will be shorter than the reference value. In the opposite case, if the apparent radius is larger than the true one (Figure 7b), we have a positive deviation; the experimental curve lies above the theoretical one, and the resulting apparent characteristic time will be longer than the reference one. An error in correctness of measure the radius of the excitation beam of 1% is high since it leads to a bias in $t_{c}$ and *D* of 5–10%.

To reduce the value of systematic error caused by incorrect measurement of the excitation beam radius, it is necessary to: (1) check the coincidence of the centers of the waist of the excitation beam and the cell, while the center of the cell may not coincide with the center of the beam waist (for more detail, see the next section); (2) find and measure the beam waist. An error of ±1 µm for a wide-focused configuration gives a bias in the fitted $t_{c}\;$ and *D* of no more than 5%. However, if $\omega_{e0}$ < 60 µm (estimated value), a bias is >5%. Even with a correct beam measurement, slight distortions of the experimental values from the theoretical results can be observed. In this case, it is necessary to have a reference sample with exactly known thermophysical parameters and measure it before the test sample.

### 3.2. Cell Positioning

The diffraction theory of the thermal-lens effect imposes a limitation on the optical path in the sample (the thin-lens approximation), as the model is valid provided the diameter of the excitation laser beam in the sample remains unchanged [78,79,80]. In our previous study, it was found that for the beam size scale used in this study, the signal linearly depends on the optical path length in the range 2–30 mm, and the precision of thermal-lens absorption measurements decreases with an increase in the path length; also, the use of cells of 40 mm or longer is inexpedient due to deviation from the model [81].

However, for shorter optical paths, a decrease in the thermal-lens signal is observed compared to a longer optical path length due to less absorption. Thus, with all other parameters being equal (cell position, irradiation power, sample, etc.), it results in lower signal-to-noise ratios. Thus, an increase in the relative standard deviation in the measured characteristic time was found in this study, which shows a deterioration in the measurement precision. For the Sudan I ethanol solution measured by the middle-focused configuration (Figure 2, Table 3), the relative standard deviation (RSD) of $t_{c}$ was 6% for a cell with *l* = 1.0 cm, while for *l* = 0.5 cm, RSD increased to 16%.

Thus, a short optical path length seems expedient from the viewpoint of better precision, but it does not lead to improved trueness of thermophysical parameters by itself, as it may increase the bias of measurements due to other sources. In fact, a shift of the cell center along the beam propagation axis from the central waist position (mispositioning) leads to a change in the signal. First, due to a larger radius of the excitation beam in the sample, the beam fluence decreases, which diminishes the signal amplitude. Secondly, a larger size of the excitation beam leads to an increase in the size of the thermal lens. Hence, it results in the distortion of the optimum mode mismatching and a change in the thermal-lens characteristic time, and there appears a bias in the measurement of thermal diffusivity. Thus, from the viewpoint of trueness, for a cell with an optical length larger than the possible value of its mispositioning (e.g., *l* = 5.0 cm, and the relative mispositioning is ca. 10–20%), the effect may be rather insignificant [81]. For 1.0 cm cells, a mispositioning of 1 mm does not result in a change in the signal of more than 5–7%, which can be considered insignificant for most measurements tasks. Thus, in this study, this path length was used in all the experiments.

**Figure 8:**
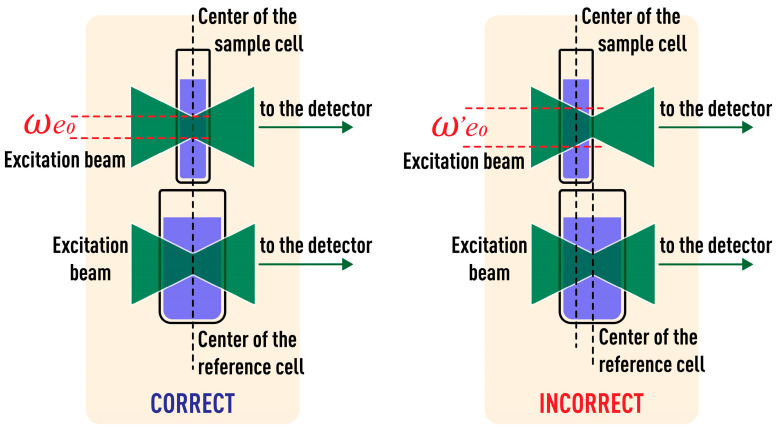
Location of sample and reference cells. See the text for details.

However, the determination of thermal diffusivity, especially in complex systems or comparison of similar samples, may require better fit of the thin-lens approach. In such a case, when the path length of the cell is commensurate with its possible mispositioning, the degree of influence will be proportional to this mispositioning and may result in unacceptable systematic errors. For instance, when a 5.0 mm cell is mispositioned relative to the excitation beam waist by 2.5 mm (50%) against its central position ($\omega_{e0}$= 42 ± 1 µm), there is different radius of the excitation beam in the center of the cell ${\omega^{\prime }}_{e0}$= 60 ± 1 µm, ${\omega^{\prime }}_{e0}$ > $\omega_{e0}$, Figure 8.

**Figure 9:**
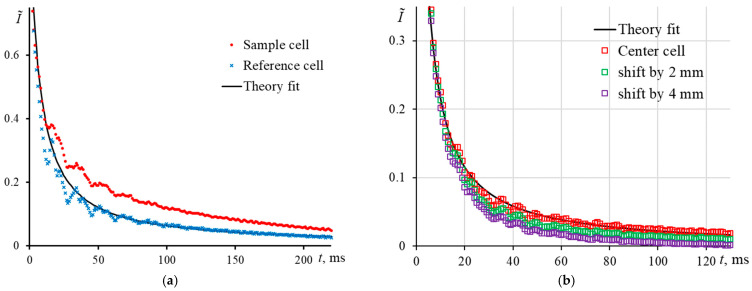
Normalized transient curves for a solution of Sudan I in ethanol (0.1 μmol/L): (**a**) 10.0 mm reference and 5.0 mm sample cells for mispositioning of 2.5 mm relative to the excitation beam waist; (**b**) a 10.0 mm sample cell for mispositioning of 2 and 4 mm relative to the excitation beam waist; middle-focused configuration ($\omega_{e0}$ = 42 µm, Figure 2, Table 3).

Normalized transient curves in this case do not correlate with the theory (Figure 9), and the measured characteristic time for the same sample was 10.1 ± 0.5 ms, while the experimental value for a correctly positioned cell was 4.95 ± 0.08 ms, which fits well with the theoretical value of 4.95. Thus, the bias of the determined characteristic time is more than 100% and, thus, unacceptable.

Thus, to minimize the bias of thermophysical measurements, the waist of the excitation beam should be in the center of the sample cell as accurately as possible; i.e., we need to obtain (i) the maximum signal indicating the central position of the excitation beam waist in the cell, and, at the same time, (ii) we need to check the shape of a transient curve and its correspondence to the theory. Certainly, a precise positioning of a sample cell can be accomplished using z-scan techniques, but this procedure is relatively time consuming and not always expedient. An alternative approach may consist in a two-cell check using a *sample* cell of a short path length that is proper for precise measurements, and a *reference* cell, with a longer path length that is used for checking the measurement bias by both the amplitude and transient signals. With the correct placement of two cells (the excitation-beam waist in the center), the experimental characteristic time will be the same, and normalized transient curves are in complete agreement with each other and the theory (Figure 8 and Figure 9). In case of differences in normalized transient curves, it is necessary to make sure that the cells are positioned correctly and, if necessary, move the sample cell along the beam propagation until the curves coincide.

### 3.3. Detector

The distance from the sample cell to the detector does not affect the shape of the transient curve and, thus, the systematic error of thermophysical measurements, but it does affect the signal. In fact, the single-point measurements in thermal lensing are based on the pinhole (or small-area) photodetectors, which integrate the central part of the probe beam. Usually, the detector is placed under Fraunhofer diffraction conditions (a far-field condition). The smaller is the pinhole, the better is the sensitivity of measurements of the relative intensity change as a result of the thermal-lens effect. However, the smaller the pinhole is, the less intensity reaches the detector, and thus, a lower signal-to-noise ratio is attained. Thus, the choice of distance from the sample to the detector pinhole is a matter of compromise. This choice is related to the divergence of the probe beam after the sample and to the size of the photosensitive area of the detector itself. The smaller the pinhole aperture, the smaller $z_{2}$ can be, and vice versa, a larger pinhole width requires a larger distance to the detector to keep the far-field conditions and a suitable signal-to-noise ratio. In the study [68], Hannachi performed an optimization of a dual-beam thermal-lens configuration and found that at $z_{cp}$ = 0.88 cm, the position $z_{2}$ < 150 cm, the random error of the thermal-lens signal is within 5–8%, and at $z_{2}$ > 150 cm, the error exceeds 15%. The author relates these changes primarily to an increase in the probe-beam size with increasing $z_{2}$, which makes it difficult to center it in the detector aperture. As a rule, the detector is installed at a distance longer than a meter from the sample. A closer location entails a decrease in the signal, since the increase in the beam radius at the detector is not large enough, and nearly all of the radiation falls on the light receiver. At the same time, a larger $z_{2}$ is also undesirable, because in this case, a small amount of radiation reaches the detector due to the beam divergence, and the appearance of thermal lens in the sample will be observed with a high noise or may even pass unnoticed.

**Figure 10:**
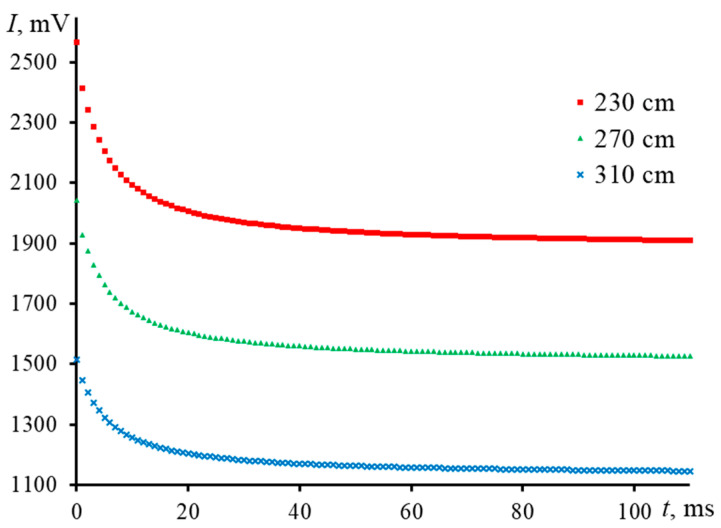
Influence of the sample-detector distance on the transient curves for an aqueous solution of ferroin (2.5 μmol/L); middle-focused configuration ($\omega_{e0}$ = 42 µm, Figure 2, Table 3).

Figure 10 shows the transient curves of an aqueous solution of ferroin taken with a middle-focused configuration ($z_{cp}$ = 2.7 mm) but with different values of $z_{2}$: 310, 270, and 230 cm. In all cases, the values of determined thermophysical parameters were the same, with similar values of the relative standard deviation (Table 4).

Since this parameter, as already mentioned, does not affect the accuracy of thermal-lens measurements of thermal diffusivity, it is impossible to strictly specify the optimal value of the sample-to-detector distance. For example, in the work of Shen et al., it is ca. 6 m [82]. For Ventura et al. [83], who analyzed biodiesel mixtures, it is ca. 4 m (at $z_{p}$ = 0.113 m); in [84] $z_{2}$ = 4.5 m ($z_{cp}$ = 1.4 cm); in [30,85], the detector was located at a distance of 0.120 and 4.04 m, respectively, from the sample ($z_{cp}$ = 0.31 for [30]). For more information about use of the sample-to-detector distance, see Table S2, the Supplementary Materials.

#### 3.3.1. Probe-Beam Spot Offset Error 

**Figure 11:**
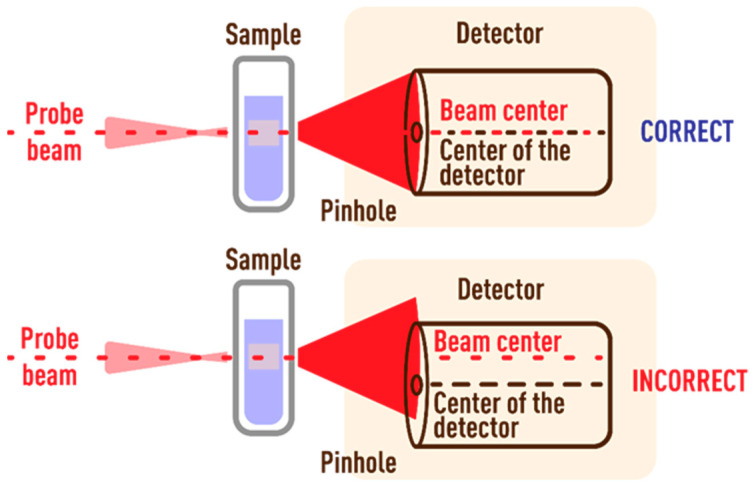
Scheme of beam incidence on the detector pinhole.

**Figure 12:**
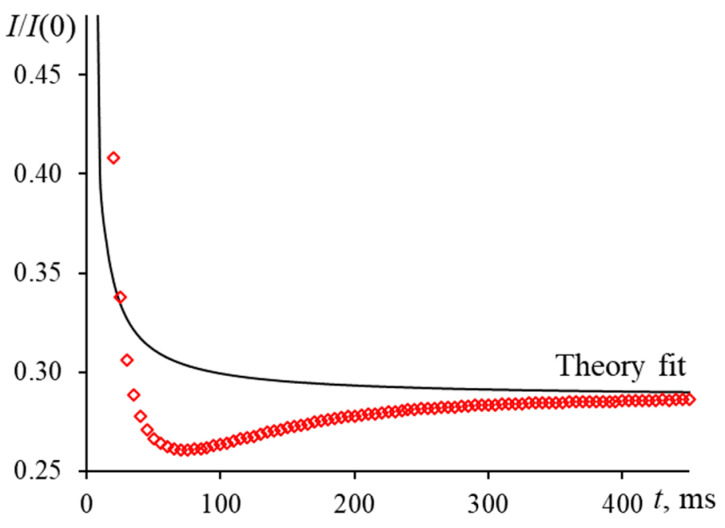
The transient curve of an aqueous solution of ferroin (2.5 μmol/L) when the intensity of the probe laser beam passing through the detector pinhole is not the maximum; narrow-focused configuration ($\omega_{e0}$ = 33 µm, Figure 1, Table 3).

An incorrect offset of the probe beam at the center of the detector introduces a high and, in some cases, unacceptable bias in determining the characteristic time. To reduce the systematic error, the center of the probe beam spot should pass through the center of the pinhole, and the detector signal is maximum (Figure 11). Small deviations of the center of the probe beam spot from the center of the detector lead to gross errors, which are visible from the shape of the transient curves. However, the signal magnitude may remain unchanged. Figure 12 shows the extreme case when the beam passes through the pinhole at the edge of the thermal lens. The shape of the time curve is similar to the time curve for systems with a pronounced Soret effect [86] or photochemical reactions [84], which can lead to false conclusions.

In such a case, the value of systematic error is unacceptable (>100%). An excellent indicator is the characteristic time function, which decreases monotonically to a minimum $I\left(t\right)$ value at about 70 ms, after which $I\left(t\right)$ increases to a theoretical value ($t_{c}$ is negative). However, this case should not be confused with thermal diffusion, which manifests itself after 300–400 ms [29,87].

Thus, to reduce the bias caused by incorrect offset of the beam on the detector, it is necessary to check the passage of the intensity maximum of the probe beam at the detector center. A check should be conducted before each measurement.

### 3.4. Sample

#### 3.4.1. The Colorant Concentration (the Distance between Molecules)

Thermal-lens measurements in the visible range are possible only for colored solutions. Dyes (colorants) are used to determine thermooptical properties of colorless samples (water, ethanol, etc.) [38,88,89,90]. The dye molecules, absorbing the radiation of the excitation laser, start the appearance of a temperature gradient field, which results in formation of a thermal lens. For the largest signal, the wavelength of the absorption maximum of the dye should be equal or close to the wavelength of the excitation laser.

In a simplified form, the equation for the probe-beam intensity can be expressed as:

(10) $$I\left(t\right)=B\left(t\right)\cdot P\cdot E\left(t\right)\cdot \alpha ,$$

where *P* is the power of the excitation laser; E is the thermooptical constant, which contains the thermophysical parameters of the system;

(11) $$E\left(t\right)=\left(-dn/dT\right)/\lambda_{e}k,$$

and α is the linear absorptivity. $B\left(t\right)$ is the geometric factor, which includes all the geometric parameters of the setup. The parameters *P* and α should not affect the shape of the transient curve. However, in some cases, usually for finely dispersed solutions, a change in the shape of the curve is seen with an increase in the absorption coefficient or irradiation power [91]. We make two assumptions about the cause of this effect. The first is the influence of solvation shells of molecular and supramolecular species; for the second, a high value of the phase shift in the propagation of the probe beam that affects the amplitude signal.

In the case of the influence of solvation shells of molecules, the absorption coefficient is α, which from Bouguer’s law determines the relation of the intensity of the transmitted light to the incident light according to the following equation:

(12) $$I=I_{0}e^{-\alpha l},$$

where I is the intensity of light transmitted through the sample; $I_{0}$ is the incident light intensity on the sample; *l* is the sample thickness (the optical path length); α is expressed as

(13) α=2.303εc,

in which, *ε* is the molar absorptivity (a constant for a given wavelength and absorbing species), and *c* is the molar concentration of the absorbing species. Thus, at a constant and precisely known value of *ε*, the linear absorption coefficient α depends on the concentration of the absorbing species in the sample. In this case, the question of concentration is a question of the distance between molecules of the absorbing substance in the selected solvent. The average distance between molecules $d_{av}$ (nm) can be estimated through a rough geometric model using the radius of a sphere according to the well-known expression,

(14) $$d_{av}\approx \left(3\cdot 10^{24}/4\pi cN_{a}\right),$$

where $N_{a}$ is the Avogadro number (6.02⋅10^23^ mol^−1^). In a simplified form, the equation can be rewritten as:

(15) $$d_{av}\approx 0.735c^{-1/3}.$$

Hence, the thermal-lens signal θ, Equation (5), at constant temperature coefficient of the refractive index dn/dT, thermal conductivity *k*, the probe wavelength, the optical path length *l*, and the excitation power *P*, is affected by the absorption coefficient or, in other words, the distance between light-absorbing molecules. In this case, according to the model [82], a change in the power and absorption coefficient should only lead to a change in the signal and not affect the time of occurrence of the thermal field. However, at the same time, at high values of α (along with signal changes), a change in the shape of the transient curve is observed, which indicates that the heat transfer mechanism changes due to the influence of both high concentration and solvated shells of absorbing molecules. This behavior was previously found for dispersed systems (fullerenes and nanodiamonds) [31,91]. At the same time, at a small value of $d_{av}$, an increase in the irradiation power leads to local overheating of the irradiated area and a change in thermal diffusivity, which is also reflected in a changed shape of the transient curve.

Since thermophysical properties are found from the shape of the transient curve, its change due to the high dye concentration in the sample leads to an error in measured characteristic time and thermal diffusivity. Of all the systematic errors, the concentration error is the most difficult to detect, since it does not manifest itself explicitly during measurements (there are no visible anomalies in the behavior of the transient curves). Hence, it is important to correctly select the optical absorption range, or, in other words, the concentration range of absorbing species, within which the shape of the transient curve of the sample still is the same.

**Figure 13:**
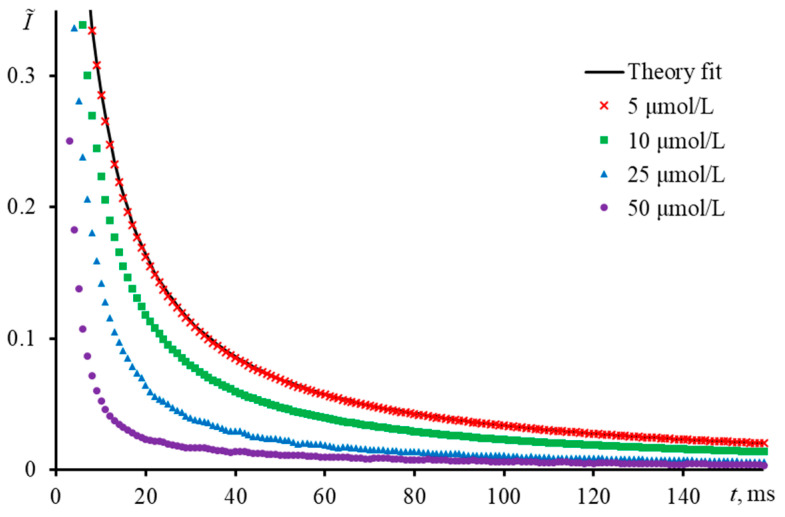
Normalized transient curves of aqueous solutions of ferroin with different concentration; narrow-focused configuration ($\omega_{e0}$ = 33 µm, Figure 1, Table 3).

By thermal lensing of a single solution, one may obtain incorrect results without notable deviations. This error can be revealed provided a series of measurements of solutions with different concentrations is conducted under the same conditions. The alert is the difference between the normalized transient curves (Figure 13). Conducting measurements on a narrow-focused configuration with aqueous ferroin, it was found that all solutions above the concentration of 5.0 μmol/L ($d_{av}$, ca. 45 nm) have different behavior in the normalized form. The indicator of different behavior is the fact that $t_{c,\;exp}\;$ and $t_{c,\;theor}$ calculated based on the known thermal and other experimental parameters are completely correlated and equal to 6.15 ms. At *c* > 5.0 μmol/L, the apparent characteristic time decreases. The largest bias in characteristic time (88%) was observed for the most concentrated solution with 50 μmol/L ($t_{c,\;exp}\;$ = 0.73 ± 0.08 ms, $d_{av}$, ca. 20 nm). The systematic error for a solution of 10.0 μmol/L was 35% ($t_{c,\;exp}\;$ = 4.05 ± 0.09 ms, $d_{av}$, ca. 34 nm). Thus, it can be concluded that an incorrectly selected concentration range of the test sample introduces an unacceptable bias in the determined thermophysical properties.

**Figure 14:**
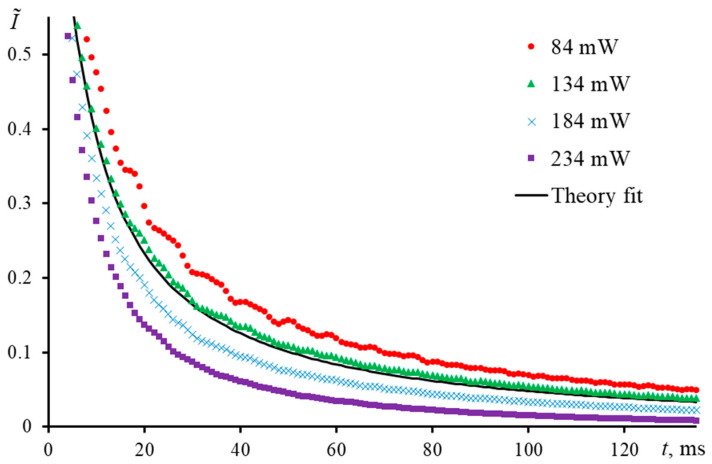
Transient curves in the normalized form of a solution of Sudan I in ethanol (2.5 μmol/L) at different excitation laser powers; middle-focused configuration ($\omega_{e0}$ = 42 µm, Figure 2, Table 3).

Another sign of the discussed effect can be the transient curves of the same sample obtained at different powers of the excitation beam. Let us consider this case by the example of the middle-focused configuration, where measurements were made of a 2.5 μmol/L Sudan I ethanol solution (Figure 14). With an increase in the excitation power from 84 to 234 mW, the curve shape changes, which leads to a change in the apparent characteristic time. This change should not be true, since there are no changes in the solution composition (phase transitions, chemical reactions, etc.). At the same time, if in the first case, where the behavior of transient curves for samples with different concentrations (obviously high) was considered, the experiment correlated with theory only in the case of very dilute solutions (< 5.0 μmol/L), then here, an experimental transient curve, which closely correlates with the theoretical one is obtained only at *P* = 134 mW. The bias in the experimental characteristic time was <1%, and it can be concluded that the experimental conditions are close to the optimum, and the spectrometer operates with high reliability. The distance between molecules in the case under consideration is ca. 90 nm. However, these conclusions are incorrect.

**Figure 15:**
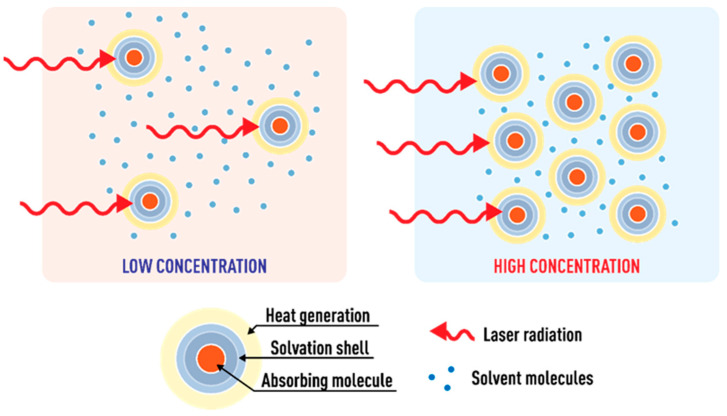
Schematic diagram of heat generation in colorant solutions at low and high concentrations.

Based on our results, the highest limit of concentration for organic solvents (in particular, for ethanol) in TLS is more than an order of magnitude lower than for water: 5.0 and 0.1 μmol/L, for water and ethanol, respectively, provided that the distances between the molecules are approximately five times higher than in water (45 and 215 nm for water and ethanol, respectively, at these concentrations). Additionally, this difference in threshold concentrations of 5.0/0.1 = 50 times is significantly larger than the ratio of thermooptical coefficients $\left(-dn/dT\right)/k\lambda_{p}$ in these media, 4.427/0.256 = 17.30. Thus, it can be concluded that the governing parameter is not the distance between the particles absorbing radiation but the amount of free solvent molecules not included in the solvation shell. In this case, heat sources shall be treated not as molecular species but as a molecule/solvation shell system. At a low dye concentration, the excited particle transfers heat to free solvent molecules in solution that are not involved in the solvation shells of radiation-absorbing molecules. In the case of an increase in concentration, fewer free solvent molecules stay in the solution and all pass into solvation shells and the mechanism of heat transfer from the excited molecule changes. The heat transfer mechanism becomes similar to heat transfer in dispersed systems (Figure 15) [31].

At the same time, it should be noted that the solvation shell of an absorbing molecule in an aqueous solution is smaller than in an organic solvent. The size of an ethanol molecule is ca. 1.7 larger than that of a water molecule (0.27 and 0.45 nm, respectively) [92]. This, as we assume, may be the reason that the concentration threshold for the ethanol solution is lower than for water and contributes to the lower threshold compared to thermooptical coefficients shown above.

Another plausible reason for the observed effects of the change in the transient curve with concentration can be a large phase shift in the propagation of the probe beam. In deriving the basic equation for the time-resolved thermal-lens curve, Shen et al. used the assumption that the phase shift that occurs as a result of changed refractive index is small ($\left|\Phi \right|\ll 1$) [70]. The phase shift depends on the change in the refractive index of the solution (Δn) according to the following equation:

(16) $$\Phi =2\pi \Delta n/\lambda_{p}\;,$$

where $\lambda_{p}$ is the probe laser wavelength. As known, the change in the refractive index Δn, in turn, depends on temperature change [70]:

(17) $$\Delta n=\Delta T\cdot \left(dn/dT\right)$$

and, thus, at constant of $\lambda_{p}$ and dn/dT, the phase shift Φ depends on the temperature change of the solution.

Following further reasoning, we can consider an equation for estimating changes in the temperature of the sample medium during a thermal-lens experiment:

(18) $$\Delta T=P\alpha /4\pi k\cdot \ln\left(1+2t/t_{c}\right),$$

where *t* is the time of generation of the thermal lens. It follows that at the moment *t*, at a constant thermal conductivity *k* and characteristic time $t_{c}$, the temperature change depends on the excitation power *P* and α, which, in turn, depends on the absorbance or concentration of the light-absorbing substance. Thus, we can conclude that the phase shift is affected by the sample absorbance and the power of the excitation laser.

Table 5 presents an estimate of the temperature change and phase shift resulting from the irradiation with an excitation laser at a thermal-lens development time of 10 ms for aqueous solutions of ferroin measured with the narrow-focused configuration. The table shows that the condition $\left|\Phi \right|\ll 1$ is satisfied for ferroin concentrations below 0.1 μmol/L, while at concentrations above 10 μmol/L, the phase shift becomes higher than 1. This may be the reason for the observed changes in the shape of the time-resolved curves at absorbances above 0.07 (Table 5 and Figure 13), while for 5 μmol/L (absorbance of 0.05) the experimental curve fits the theory. Thus, from the viewpoint of the phase shift, we can propose using colorant concentrations providing a phase shift below 1.

Comparing both plausible causes—the solvation-shell effect at close intermolecular distances and the overall phase shift—they seem to act together. The estimation of the concentration threshold of the solvation shells is coincident with that for the phase shift (5 μmol/L of ferroin in water). In our opinion, this is due to the impact of both the total temperature increase at high concentrations (Table 5) and changes in the heat transfer rate at the early thermal-lens development due to the appearing heterogeneity of the system (intermolecular distances). However, this phenomenon is complex and should be considered in more detail in a separate study, which was beyond the frames of this paper.

The remark on the total temperature increase is also valid for selecting the excitation power for an experiment with a constant colorant concentration: a decrease in the apparent characteristic time is also observed with an increase in the excitation laser power. This also agrees well with the total increase in heat transfer for higher powers and the appearance of a heterogeneous thermal field upon higher heating of individual heat sources (colorant solvated molecules).

Nevertheless, from a practical point of view, to eliminate the bias caused by the high concentration of the absorbing species in the solution, it is necessary to determine the concentration range. To accomplish this, it is necessary to measure a series of 4–5 solutions with different concentrations but under the same conditions and compare the normalized transient curves. If all curves show the same behavior, then the measurement can be conducted on any solution. Additionally, a colorant concentration of 5 µmol/L for aqueous and 0.1 µmol/L for organic solvents should not be exceeded. If the concentration of the colorant is not sufficient to detect TLS, it is necessary to increase the power of the excitation beam.

#### 3.4.2. Microimpurities and Dust

**Figure 16:**
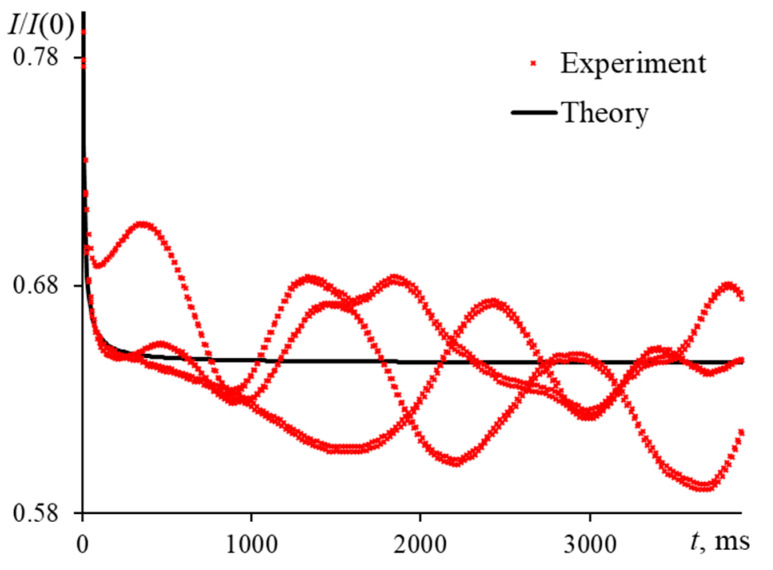
Fluctuations in individual transient curves for a ferroin solution (2.5 μmol/L) attributed to the presence of dust; narrow-focused configuration ($\omega_{e0}$ = 33 µm, Figure 1, Table 3).

The presence of microimpurities, random pollutants, and/or dust in the samples under study strongly affects the behavior of the thermal lens. This presence entails the appearance of noise as pronounced deviations on the transient curves. This is demonstrated in Figure 16, which shows measurements of an aqueous solution of ferroin at a concentration of 2.5 μmol/L on a narrow-focused configuration (Figure 1, Table 3). Dust and fine particles cause chaotic fluctuations on the transient curve. However, when assessing thermal diffusivity, the calculations are based on the parameters of the averaged transient curves. When averaging is made for a large set of curves (more than 200), and the frequency of fluctuations is low, the systematic error is insignificant. However, differently from this, averaging less than 20 curves introduces a large bias (>10%) and is clearly seen in reaching the steady state and in the characteristic time.

As described in [93], the time required to reach the steady state increases with *m* (for *m* of ca. 50, t  > 100$t_{c}\;$). In our case (an aqueous solution), $t_{c,\;theor}$ = 6.15 ms (Figure 16). However, in the experiment, the steady state is not reached even after 4 s of development. A similar behavior of thermal-lens measurements is observed for some coarse dispersions. In our case, dust microparticles are present in the sample. A similar picture of fluctuations is observed in [94], where the analysis of CdSe nanoparticles with sizes of 4.6–5.1 nm was carried out.

Therefore, to reduce the bias associated with the presence of coarse dispersed particles in solution, it is necessary to measure freshly prepared samples using pure solvents. Furthermore, before thermal-diffusivity measurements, it is necessary to filter the analyzed sample. This is contrary to chemical analysis problems, when the analyte concentration may be decreased, this will not affect the thermophysical properties of the sample. The choice of filter membrane material and pore size in this case is different in each case and depends on the solvent and the analyzed sample [95,96,97]. An example of transition curves for a filtered and unfiltered ferroin aqueous solution is shown in Figure S1.

### 3.5. Time to Reach a Steady-State Thermal Lens

#### 3.5.1. Shutter Frequency

To find the characteristic time and thermal diffusivity, we used the full development of the thermal field and the transition of the transient curve to a stationary state. The larger the diameter of the excitation beam (as well as the factor *m*), the longer it takes the signal to reach the steady state (as previously established for the dual-beam thermal-lens spectrometers with a high value of *m,* t  > 100$t_{c}\;$ [93]) [66]. Working under such conditions at high shutter frequencies may be impractical due to a failure to reach a steady state. A compromise in the case of a narrow-focused configuration is reached at *m* ≈ 4. This made it possible to quickly reach the thermal equilibrium of the thermal lens and increase the sensitivity. Here, we will not consider the time needed for the relaxation of the thermal lens and the time ratio of the open and closed states of the shutter. Based on the data of [98], a 50/50 ratio of the open and closed states of the shutter was decided.

**Figure 17:**
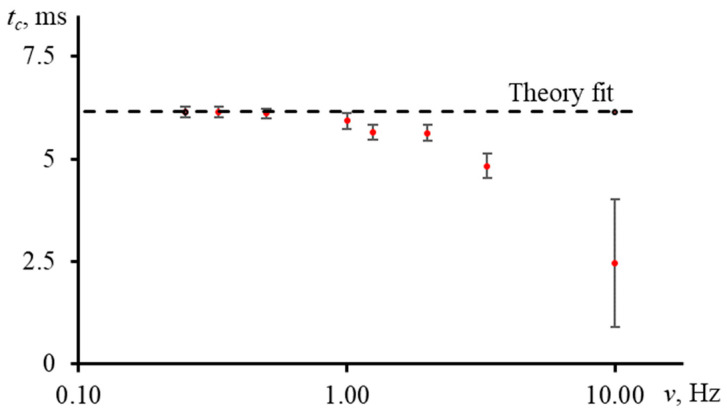
Characteristic time for an aqueous solution of ferroin (2.5 μmol/L) as a function of the modulation frequency; narrow-focused configuration ($\omega_{e0}$ = 33 µm, Figure 1, Table 3).

Figure 17 shows the result of measuring the characteristic time of an aqueous ferroin (2.5 μmol/L) for a narrow-focused configuration (Figure 1, Table 3) at different shutter frequencies.

**Figure 18:**
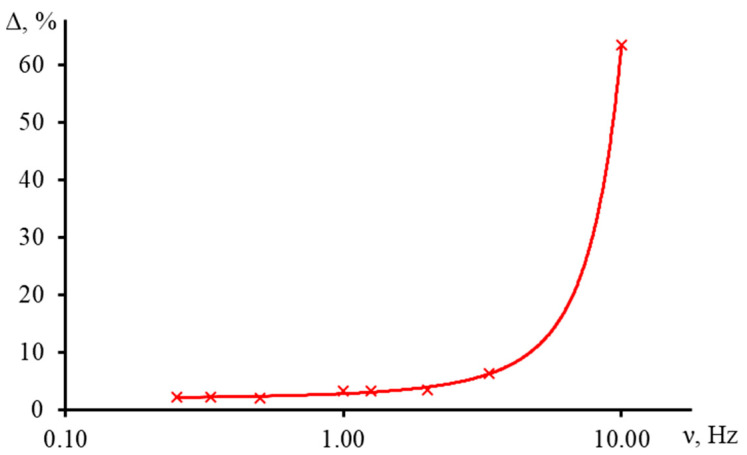
Measurement error of the characteristic time on the modulation frequency of an aqueous solution of ferroin (2.5 μmol/L); narrow-focused configuration ($\omega_{e0}$ = 33 µm, Figure 1, Table 3).

As can be seen, as the shutter frequency increases, the systematic error in finding the characteristic time increases (Figure 18). At a frequency of 1 Hz, the difference between the experimental and theoretical $t_{c}$ was within 4%. This bias has a moderate effect on the correctness of finding the thermal diffusivity. At lower frequencies, this difference decreases, and the experiment fully agrees with the theory. On the other hand, at higher frequencies, the curve does not have time to reach the steady state, which leads to a decrease in the trueness in the determined $t_{c}$ (at a frequency of 10 Hz, the bias was more than 60%, Figure 18).

To reduce the value of systematic error of thermophysical parameters due to the incorrectly selected modulator frequency, it is necessary to conduct a series of measurements of a reference sample at a different frequency and find the boundary conditions. For used setups, frequencies below 2 Hz are best for common types of solvents (water, ethanol, toluene, acetonitrile, etc.).

#### 3.5.2. Mode-Mismatch Factor

In this section, the influence of the mode-mismatch factor *m* on the trueness of measurement the thermophysical parameters is considered; the provisions of Shen’s theoretical model will not be disputed. Let us consider the operation of a narrow-focused configuration of a thermal-lens spectrometer (Figure 1 and Table 3). Shen’s model predicts that a thermal-lens spectrometer with a large mode-mismatch factor has a high sensitivity and therefore is recommended for use in chemical analysis. Thus, a dual-beam thermal-lens spectrometer with a mode-mismatch factor fully exploits the change in the refractive index of the medium when *m* is large (*m* > 10) [82]. 

**Figure 19:**
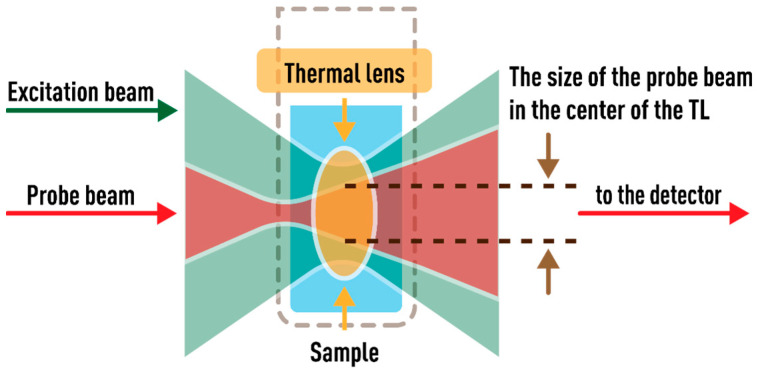
Scheme of beam propagation through the sample at a mode-mismatch factor less than 1.

At *m* < 1, the thermal lens is several times larger than the radius of the probe beam (Figure 19), which leads to a change in the diffraction pattern [77], a change in the course of the transient curve, and, thus, incorrect results in the determined thermal diffusivity.

**Figure 20:**
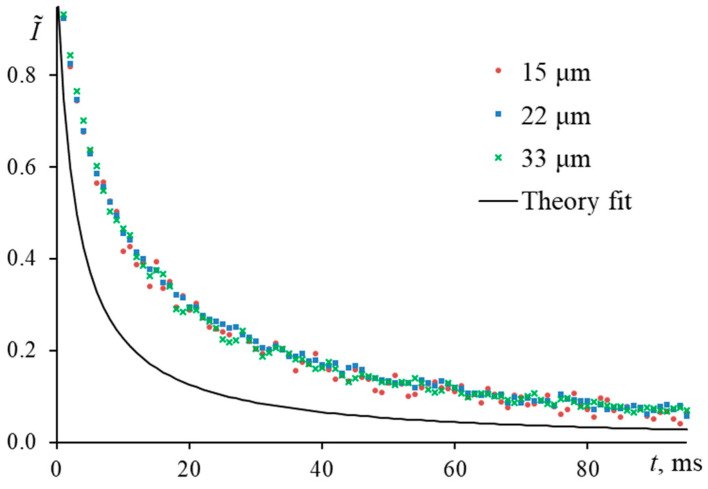
Normalized transient curves at the mode-mismatch factor *m* ≤ 1 for an aqueous solution of ferroin (2.5 μmol/L); narrow-focused configuration ($\omega_{e0}$ = 33 µm, Figure 1, Table 3).

Figure 20 shows the normalized transient curves of solutions of ferroin in water (2.5 μmol/L) for a case when the probe beam radius is less than or equal to the excitation beam radius (15 ± 1, 22 ± 1, and 33 ± 1 µm). Visually, this is akin to a case when there is an incorrect measurement in the radius of the excitation beam; namely, the measured radius of the excitation beam is larger than the true one. The found characteristic time is more than twofold higher than the theoretical value ($t_{c,\;exp}$ = 4.7 ± 0.8 ms, $t_{c,\;theor}$ = 1.9 ms). The experimental curves in the normalized form agree with each other, which shows that the other experimental conditions are correct. However, the theoretical curve modeled for this case seriously differs from the experiment.

**Figure 21:**
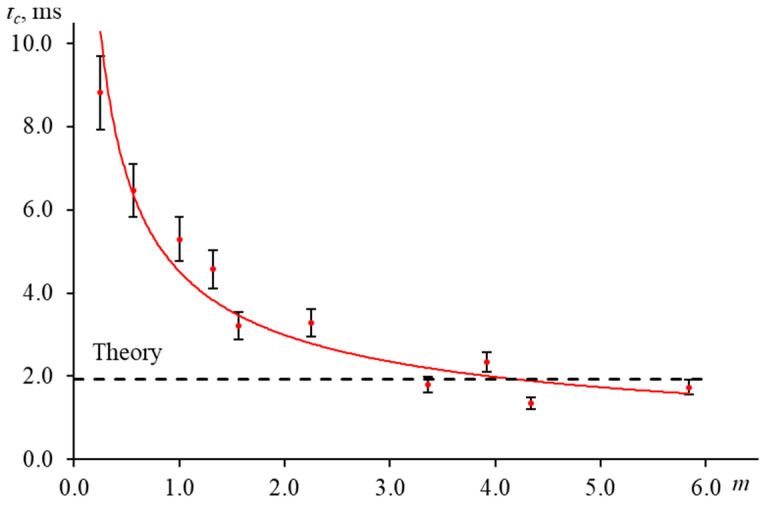
Behavior of the characteristic time for an aqueous solution of ferroin (2.5 μmol/L) with increasing mode-mismatch factor *m*; narrow-focused configuration ($\omega_{e0}$ = 33 µm, Figure 1, Table 3).

As $\omega_{p1}\;$ and *m* increase, the experimental transient curves and the theoretical ones converge. The finding characteristic time (Figure 21) then decreases and approaches the calculation. Thus, at *m* = 5.8, the deviation of the experimental $t_{c}$ from the theory was 11%, while at *m* < 1 it was two or more times higher than the calculation. In both cases, the value of systematic error is unacceptable.

To reduce the systematic error due to the mode mismatch factor, it is necessary to strictly observe the conditions of applicability of Shen’s model, where the mode mismatch factor is greater than 2. In this case, we have quantitatively demonstrated how the determination of the thermal diffusivity will be affected at *m* < 2 [70]. To reduce or avoid the bias due to an incorrectly selected mode-mismatch factor, it is necessary to recheck the sizes of the beams in the center of the cell. At the same time, based on our observations, it seems expedient rechecking the beam sizes at least every 3 months.

#### 3.5.3. Measurement Time on the Accuracy of the Characteristic Time

**Figure 22:**
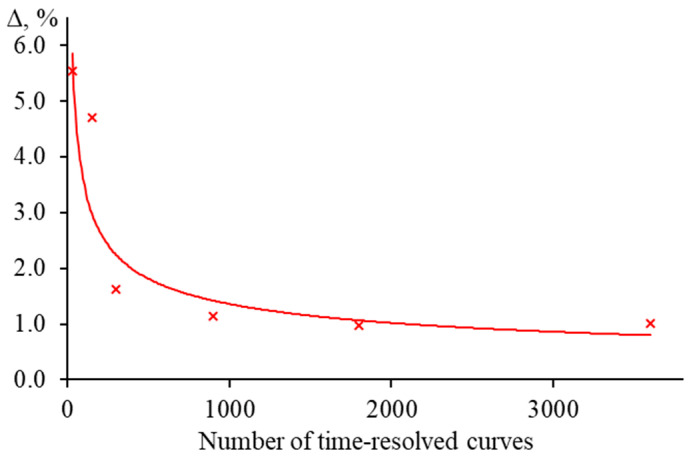
Change in the measurement error of the characteristic time from the number of averaged transient curves on the example of a solution of Sudan I in ethanol (0.1 μmol/L); middle-focused configuration ($\omega_{e0}$ = 42 µm, Figure 2, Table 3).

Increasing the number of transient curves for averaging leads to better trueness of the characteristic time and thermal diffusivity vales but increases the analysis time. Hence, it is necessary to find a balanced number of time-resolved curves to find $t_{c}$ and *D* with a given accuracy. Let us consider the influence (Figure 22) of this factor on the example of a middle-focused configuration with a modulator frequency of 1 Hz (Figure 2, Table 3), using a solution of Sudan I in ethanol (0.1 μmol/L). When averaging 30 transient curves (1 min of measurements), the systematic error in determining $t_{c}$ was above 5%, and when averaging 3600 (two hours of continuous measurements), it decreased to 1%. At the same time, with an increase in the time of the experiment and the number of time-resolved curves, the value of random error decreases. In the first case, $t_{c,\;exp}\;$ was 5.22 ± 0.20 ms (RSD = 3.8%), and in the second case,$\;t_{c,\;exp}\;$ = 4.90 ± 0.09 ms (RSD = 1.8%), while the theoretical value is 4.95 ms. A further increase in the transient curves (increase in the analysis time) has an insignificant effect on the accuracy of $t_{c}$ and *D.*

The selection of the number of averaged transient curves and, consequently, the measurement duration depends on the task at hand. To estimate the thermophysical values of a sample, the required number of averaged curves is small, and it will be sufficient to average the first 10–12 min of the experiment (300–400 curves). To solve research problems where high accuracy of the obtained values is needed, long-term measurements and averaging of a large set of transient curves are necessary (experiment time is more than an hour, the number of averaged transient curves is above 1000).

### 3.6. Summary

**Figure 23:**
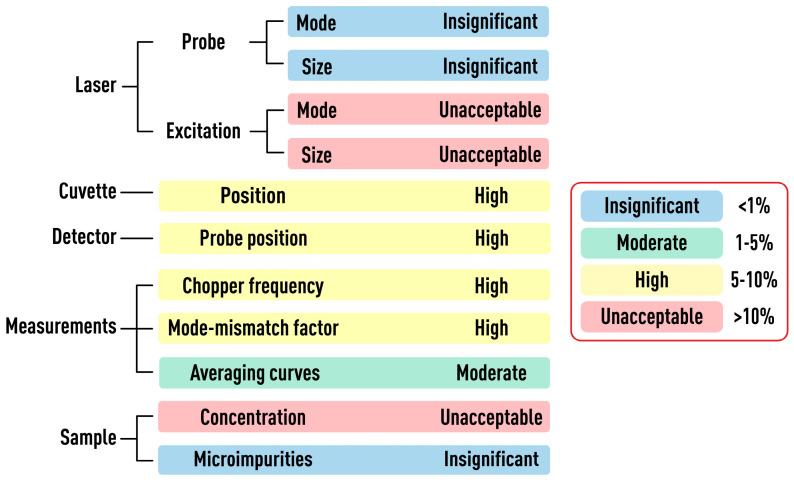
Evaluation of the impact of systematic error sources on the trueness in thermophysical properties by thermal-lens spectrometry.

Below is a summary diagram of the influence of each considered factor on the trueness of the assessment characteristic time and thermal diffusivity by dual beam thermal lensing (Figure 23). Within the framework of the setup configurations considered, several conclusions can be drawn, which are summed up and outlined below. The largest value of the systematic error of thermophysical parameters is introduced by the following factors: the changes of the transverse spatial mode and waist radius of the excitation laser and the concentration range of the sample. Any change in the spatial mode of the excitation beam leads to a change in the size and position of the beam waist, which adversely affects the reliability of the found thermal diffusivity. If short-term changes (within a few hours) are relatively easy to detect, long-term changes (with a period of a day or more) are more difficult to detect, and, at the same time, they introduce the largest systematic error. In our case, changes in the beam waist of more than 20% with a day-long period were revealed, which led to the bias in the measurement of the characteristic time of 50%, which is unacceptable for any measurement objectives.

Another major source of the systematic error is the concentration range of the analyzed sample. This error is detected either when measuring a series of samples with different concentrations or if the sample at different irradiation power shows a change in the shape of the transient curve (as we suppose, within a change of about 200 mW). The systematic error in the narrow-focused configuration reached more than 80%.

Factors such as the sample-cell position, the probe beam positioning on the detector, the modulation frequency, and the mode-mismatch factor have a high influence on the trueness of the thermal-lens characteristic time. In all cases, the systematic error of thermal diffusivity ranges from 5 to 10%, which can be considered as high both for the characterization of thermal materials such as nanofluids and for chemical analysis. Concerning the cell positioning, a systematic error is negligible when using a cell with an optical path length greater than the possible mispositioning value (along the beam propagation). However, if the size of the cell is close to its possible misplacement values, the impact would be proportional to this misplacement and in extreme cases can reach 100%.

Incorrect positioning of the probe beam on the detector, in extreme cases, can also introduce an unacceptable systematic error. However, if we consider the case when the detector pinhole is within the boundaries of the appearance and dissipation of the thermal lens, the bias in assessing the characteristic time $t_{c}\;$ is up to 10% (depending on the shift value). This value can be reduced by checking the position of the maximum intensity of the probe in the detector center before each measurement, at least once a day.

The trueness of the characteristic time depends on the modulation frequency and as a result, on the possibility to reach the steady state. For an excitation beam waist radius $\omega_{e0}$ = 33 μm and the influence on the signal rise in an aqueous solution, we found that at a frequency of 1 Hz, the bias in the characteristic time reached no more than 4%, and a tenfold increase in frequency resulted in a 60% error.

The last factor of a high value of systematic error is an incorrectly selected mode-mismatch factor. With a narrow-focused spectrometer configuration with a mode-mismatch factor *m* < 1, the characteristic time differs by more than two times from the theory. Only when *m* > 3, the value of $t_{c}\;$ becomes close to the theoretical value.

Changes in the spatial mode, as well as the incorrect measurement of beam radius for the probe laser, have low effect on the determination of the characteristic time $t_{c}\;$ and thermal diffusivity *D*. An error of 5% for the probe beam radius in the cell introduces the bias of <1% in determination of characteristic time and thermal diffusivity. Among the factors considered, random fluctuations caused by the presence of a dispersed phase in the form of dust in the solution also do not have a significant effect on $t_{c}$ and *D*. Averaging a big set of curves (300–400) leads to smoothing of all fluctuations and does not influence the characteristic time significantly. However, another problem follows from this: the incorrectly selected number of averaged curves. Here, the systematic error of thermophysical parameters is 1–5% (we omit the extreme cases when averaging is conducted over 2–10 curves and the error can reach 30–40%) and introduces a moderate error. According to our data, at a frequency of 1 Hz, the best number of averaged curves is 300–400 (which corresponds to 10–12 min of measurements). The bias under these conditions did not exceed 5%. A further increase in the number of averaged curves to 3600 (two hours of measurements) decreased the systematic error of assessing $t_{c}$ and *D* to 1 %.

Thus, the frequency of checking the operation of the main components and measurement parameters is given to reduce value of systematic errors associated with incorrect operation of the optical components of a dual-beam mode-mismatch configuration:

1. Check of lasers for stability in divergence (at least once per month).
2. Check of the radius of the beams in the cell (at least once per month).
3. Check of coincidence of the centers of the excitation and probe beams (1.5–2 months).
4. Check of the location of the excitation beam in the center of the cell with the sample (at least once every three months).
5. Check of the location of the center of intensity of the probe beam at the center of the detector at least once every three months).
6. Sample measurements (customized): checking the concentration range; working out the required frequency of the shutter; working off the measurement time and the number of transient curves for averaging.

The results presented in this work help to identify and reduce the systematic errors of thermal diffusivity measurements (caused by various sources) and may be useful for all kinds of research involving the applications of thermal-lens spectrometry.

## 4. Conclusions

In conclusion, the problem of the systematic error in determination of thermophysical parameters by thermal-lens spectrometry is significant. The paper considers a large number of possible sources of trueness present in the measurement of the characteristic time and thermal diffusivity of liquid samples by TLS in the dual-beam version. Among the factors considered, some were identified as insignificant, since their contribution to the value of systematic error can be neglected, such as in the case of changes in the radius of the probe beam. At the same time, the systematic error of most factors can be reduced to a low or even negligible level, including cell, detector, and measurement parameters as well as effects of microimpurities in the sample. An exception is the unstable operation of the excitation laser, causing substantial changes in the beam itself (divergence and waist radius). Such changes must be considered each time when measuring thermophysical properties and adjusted for (correctly selecting the measurement time range). The question of the concentration range of the sample stands apart, since it requires separate consideration for each system of absorbing particle–solvent. However, as a conclusion and as a rule, it can be claimed that one should not go beyond the colorant concentrations of 1 and 0.1 μmol/L for aqueous and organic systems, respectively.

## Figures and Tables

**Table 1:** Excitation laser parameters; laser model MGL-FN.

Property	Value
Wavelength, nm	532.0 ± 1.0
Radiation power, mW	(10.0–500.0) ± 0.3
Polarization	>100:1
Mode, TEM_00_	>95%

**Table 2:** Probe laser parameters; laser model HNL050L.

Property	Value
Wavelength, nm	632.8
Divergence of the beam in the cell	<1%
Radiation power, mW	5.00 ± 0.01
Polarization	>500:1
Mode, TEM_00_	>95%

**Table 3:** Setup parameters in thermal-lens experiments (*n* = 10, *P* = 0.95).

Parameter	Configuration		
	Narrow-Focused	Middle-Focused	Wide-Focused
Focal length of the excitation laser lens (*f_e_*), mm	200.0	300.0	400.0
Radius of the excitation laser in the waist ($\omega_{e0}$), µm	33.0 ± 1.0	42.0 ± 1.0	82.0 ± 1.0
Rayleigh length for excitation laser ($z_{ce}$), mm	6.6 ± 0.5	10.4 ± 0.5	39.7 ± 1.0
Focal length of the probe laser lens (*f_p_*), mm	200.0	300.0	
Radius of the probe laser in the waist ($\omega_{p0}$), µm	14.5 ± 0.5	23.5 ± 0.5	
Rayleigh length for probe laser ($z_{cp}$), mm	1.0 ± 0.5	2.7 ± 0.5	
Radius of the probe laser in the cell ($\omega_{p}$), µm	60.0 ± 1.0	60.0 ± 1.0	100 ± 1.0
Distance from laser to sample for probe laser, m	1.0	4.2	
Distance from laser to sample for excitation laser, m	0.7	1.2	
Sample-detector distance ($z_{2}$), m	0.60	2.3–3.1	
Excitation laser power, mW	20.0 ± 0.3	200.0 ± 0.3	
Probe laser power, mW	4.95 ± 0.01	7.10 ± 0.01	
Shutter frequency, Hz	1		
Cell length, mm	10.00 and 5.00 ^1^		

^1^ Applied in Section 3.2.

**Table 4:** Thermophysical parameters of a 2.5 μmol/L aqueous solution of ferroin (*n* = 5, *P* = 0.95).

*Z*_2_, cm	$t_{c},\;\mathrm{ms}$	RSD, %	*D*, mm^2^/s	RSD, %
230	3.12 ± 0.05	1.6	0.141 ± 0.004	2.8
270	3.03 ± 0.07	2.3	0.145 ± 0.004	2.8
310	3.13 ± 0.07	2.2	0.141 ± 0.003	2.1

**Table 5:** Estimation of the phase shift for aqueous solutions of ferroin at a constant power of the excitation laser (20 mW) for the narrow-focus configuration ($\omega_{e0}$ = 33 µm, Figure 1, Table 3).

Ferroin Concentration (μmol/L)	*A*	ΔT, K	$\left|\Phi \right|$
0.01	0.00011	0.0002	0.0015
0.1	0.0011	0.002	0.015
1	0.011	0.02	0.15
5	0.055	0.08	0.74
10	0.073	0.11	0.98
25	0.190	0.28	2.55
50	0.395	0.59	5.30

## Data Availability

Not applicable.

## Supplementary Materials

The following Supplementary Materials can be downloaded at: https://www.mdpi.com/article/10.3390/nano13030430/s1, Table S1. Nomenclature of thermal-lens measurements. Table S2. Examples of geometric parameters of spectrometers for dual-beam TLS in the far field, where measurements are carried out according to the Shen–Snook model. Figure S1. Transient curves for filtered and non-filtered aqueous solution of ferroin (2.5 μmol/L); narrow-focused configuration ($\omega_{e0}$ = 33 µm, Figure 1, Table 3).

## References

[1] Attia A.B.E., Balasundaram G., Moothanchery M., Dinish U.S., Bi R., Ntziachristos V., Olivo M. (2019). A review of clinical photoacoustic imaging: Current and future trends. Photoacoustics.

[2] Jeon S., Kim J., Lee D., Baik J.W., Kim C. (2019). Review on practical photoacoustic microscopy. Photoacoustics.

[3] Bialkowski S.E., Astrath N.G.C., Proskurnin M.A. (2019). Photothermal Spectroscopy Methods.

[4] Franko M., Tran C.D. (2010). Thermal Lens Spectroscopy. Encyclopedia of Analytical Chemistry.

[5] Proskurnin M.A., Khabibullin V.R., Usoltseva L.O., Vyrko E.A., Mikheev I.V., Volkov D.S. (2022). Photothermal and optoacoustic spectroscopy: State of the art and prospects. Phys. Uspekhi.

[6] Franko M., Goljat L., Liu M., Budasheva H., Zorz Furlan M., Korte D. (2023). Recent Progress and Applications of Thermal Lens Spectrometry and Photothermal Beam Deflection Techniques in Environmental Sensing. Sensors.

[7] Deus W.B., Ventura M., Silva J.R., Andrade L.H.C., Catunda T., Lima S.M. (2019). Monitoring of the ester production by near-near infrared thermal lens spectroscopy. Fuel.

[8] Constantino R., Lenzi G.G., Franco M.G., Lenzi E.K., Bento A.C., Astrath N.G.C., Malacarne L.C., Baesso M.L. (2017). Thermal Lens Temperature Scanning technique for evaluation of oxidative stability and time of transesterification during biodiesel synthesis. Fuel.

[9] Franko M., van de Bovenkamp P., Bicanic D. (1998). Determination of trans-beta-carotene and other carotenoids in blood plasma using high-performance liquid chromatography and thermal lens detection. J. Chromatogr. B Biomed. Sci. Appl..

[10] Luterotti S., Franko M., Bicanic D. (2002). Fast quality screening of vegetable oils by HPLC-thermal lens spectrometric detection. J. Am. Oil Chem. Soc..

[11] Martelanc M., Ziberna L., Passamonti S., Franko M. (2016). Application of high-performance liquid chromatography combined with ultra-sensitive thermal lens spectrometric detection for simultaneous biliverdin and bilirubin assessment at trace levels in human serum. Talanta.

[12] Sato K., Yamanaka M., Hagino T., Tokeshi M., Kimura H., Kitamori T. (2004). Microchip-based enzyme-linked immunosorbent assay (microELISA) system with thermal lens detection. Lab Chip.

[13] Cassano C.L., Mawatari K., Kitamori T., Fan Z.H. (2014). Thermal lens microscopy as a detector in microdevices. Electrophoresis.

[14] Proskurnin M.A., Bendrysheva S.N., Ragozina N., Heissler S., Faubel W., Pyell U. (2005). Optimization of instrumental parameters of a near-field thermal-lens detector for capillary electrophoresis. Appl. Spectrosc..

[15] Nedosekin D.A., Bendrysheva S.N., Faubel W., Proskurnin M.A., Pyell U. (2007). Indirect thermal lens detection for capillary electrophoresis. Talanta.

[16] Shokoufi N., Shemirani F. (2007). Laser induced-thermal lens spectrometry after cloud point extraction for the determination of trace amounts of rhodium. Talanta.

[17] Shokoufi N., Hamdamali A. (2010). Laser induced-thermal lens spectrometry in combination with dispersive liquid-liquid microextraction for trace analysis. Anal. Chim. Acta.

[18] Andrade A.A., Coutinho M.F., de Castro M.P.P., Vargas H., Rohling J.H., Novatski A., Astrath N.G.C., Pereira J.R.D., Bento A.C., Baesso M.L. (2006). Luminescence quantum efficiency investigation of low silica calcium aluminosilicate glasses doped with Eu_2_O_3_ by thermal lens spectrometry. J. Non-Cryst. Solids.

[19] Figueiredo M.S., Santos F.A., Yukimitu K., Moraes J.C.S., Silva J.R., Baesso M.L., Nunes L.A.O., Andrade L.H.C., Lima S.M. (2013). Luminescence quantum efficiency at 1.5 μm of Er3+-doped tellurite glass determined by thermal lens spectroscopy. Opt. Mater..

[20] Yu F., Kachanov A.A., Koulikov S., Wainright A., Zare R.N. (2009). Ultraviolet thermal lensing detection of amino acids. J. Chromatogr. A.

[21] Ventura M., Silva J.R., Catunda T., Andrade L.H.C., Lima S.M. (2021). Identification of overtone and combination bands of organic solvents by thermal lens spectroscopy with tunable Ti: Sapphire laser excitation. J. Mol. Liq..

[22] Silva J.R., Andrade L.H.C., Lima S.M., Guyot Y., Giannini N., Sheik-Bahae M. (2019). Investigation of allowed and forbidden electronic transitions in rare earth doped materials for laser cooling application by thermal lens spectroscopy. Opt. Mater..

[23] Ventura M., Silva J.R., Andrade L.H.C., Scorza Junior R.P., Lima S.M. (2018). Near-near-infrared thermal lens spectroscopy to assess overtones and combination bands of sulfentrazone pesticide. Spectrochim. Acta A Mol. Biomol. Spectrosc..

[24] Colcombe S.M., Lowe R.D., Snook R.D. (1997). Thermal lens investigation of the temperature dependence of the refractive index of aqueous electrolyte solutions. Anal. Chim. Acta.

[25] Colcombe S.M., Snook R.D. (1999). Thermal lens investigation of the temperature dependence of the refractive index of organo–aqueous solutions. Anal. Chim. Acta.

[26] Proskurnin M.A., Chernysh V.V., Pakhomova S.V., Kononets M.Y., Sheshenev A.A. (2002). Investigation of the reaction of copper(I) with 2,9-dimethyl-1,10-phenanthroline at trace level by thermal lensing. Talanta.

[27] Ventura M., Deus W.B., Silva J.R., Andrade L.H.C., Catunda T., Lima S.M. (2018). Determination of the biodiesel content in diesel/biodiesel blends by using the near-near-infrared thermal lens spectroscopy. Fuel.

[28] Arnaud N., Georges J. (2004). Cw-laser thermal lens spectrometry in binary mixtures of water and organic solvents: Composition dependence of the steady-state and time-resolved signals. Spectrochim. Acta A Mol. Biomol. Spectrosc..

[29] Arnaud N., Georges J. (2001). Investigation of the thermal lens effect in water-ethanol mixtures: Composition dependence of the refractive index gradient, the enhancement factor and the Soret effect. Spectrochim. Acta A Mol. Biomol. Spectrosc..

[30] Kononets M.Y., Proskurnin M.A., Bendrysheva S.N., Chernysh V.V. (2001). Investigation of adsorption of nanogram quantities of iron(II) tris-(1,10-phenanthrolinate) on glasses and silica by thermal lens spectrometry. Talanta.

[31] Mikheev I.V., Usoltseva L.O., Ivshukov D.A., Volkov D.S., Korobov M.V., Proskurnin M.A. (2016). Approach to the Assessment of Size-Dependent Thermal Properties of Disperse Solutions: Time-Resolved Photothermal Lensing of Aqueous Pristine Fullerenes C60and C70. J. Phys. Chem. C.

[32] Almeida A.S., Rivera G., Sousa C.A., Santos F.E.P., Souza D.N. (2019). Thermal lens spectroscopy dosimetry at high doses using a commercial transparent glass. Radiat. Meas..

[33] Shahriari E., Varnamkhasti M.G., Zamiri R. (2015). Characterization of thermal diffusivity and optical properties of Ag nanoparticles. Opt. Int. J. Light Electron Opt..

[34] Sampaio J.A., Gama S., Baesso M.L., Catunda T. (2005). Fluorescence quantum efficiency of Er3+ in low silica calcium aluminate glasses determined by mode-mismatched thermal lens spectrometry. J. Non-Cryst. Solids.

[35] Brennetot R., Georges J. (1998). Pulsed-laser mode-mismatched dual-beam thermal lens spectrometry: Comparison of the time-dependent and maximum signals with theoretical predictions. Spectrochim. Acta Part A Mol. Biomol. Spectrosc..

[36] Lopes C.S., Lenart V.M., Turchiello R.F., Gómez S.L. (2018). Determination of the Thermal Diffusivity of Plasmonic Nanofluids Containing PVP-Coated Ag Nanoparticles Using Mode-Mismatched Dual-Beam Thermal Lens Technique. Adv. Condens. Matter Phys..

[37] Gutierrez Fuentes R., Pescador Rojas J.A., Jiménez-Pérez J.L., Sanchez Ramirez J.F., Cruz-Orea A., Mendoza-Alvarez J.G. (2008). Study of thermal diffusivity of nanofluids with bimetallic nanoparticles with Au(core)/Ag(shell) structure. Appl. Surf. Sci..

[38] Saavedra R., Soto C., Gómez R., Muñoz A. (2013). Determination of lead(II) by thermal lens spectroscopy (TLS) using 2-(2’-thiazolylazo)-p-cresol (TAC) as chromophore reagent. Microchem. J..

[39] Lima S.M., Steimacher A., Medina A.N., Baesso M.L., Petrovich M.N., Rutt H.N., Hewak D.W. (2004). Thermo-optical properties measurements in chalcogenide glasses using thermal relaxation and thermal lens methods. J. Non-Cryst. Solids.

[40] Abbas Ghaleb K., Georges J. (2004). Investigation of the optimum optical design for pulsed-laser crossed-beam thermal lens spectrometry in infinite and finite samples. Spectrochim. Acta A Mol. Biomol. Spectrosc..

[41] Qiu L., Ouyang Y., Li F., Qiu L., Feng Y. (2022). Experimental Techniques Overview. Micro and Nano Thermal Transport.

[42] Proskurnin M.A., Volkov D.S., Gor’kova T.A., Bendrysheva S.N., Smirnova A.P., Nedosekin D.A. (2015). Advances in thermal lens spectrometry. J. Anal. Chem..

[43] Usoltseva L.O., Korobov M.V., Proskurnin M.A. (2020). Photothermal spectroscopy: A promising tool for nanofluids. J. Appl. Phys..

[44] Mathew S., Francis F., Joseph S.A., Kala M.S. (2021). Enhanced thermal diffusivity of water based ZnO nanoflower/rGO nanofluid using the dual-beam thermal lens technique. Nano-Struct. Nano-Objects.

[45] Raj V., Swapna M.S., Kumar K.S., Sankararaman S. (2020). Time series analysis of duty cycle induced randomness in thermal lens system. Optik.

[46] Ramírez J.F.S., Pérez J.L.J., Valdez R.C., Orea A.C., Fuentes R.G., Herrera-Pérez J.L. (2006). Thermal Diffusivity Measurements in Fluids Containing Metallic Nanoparticles using Transient Thermal Lens. Int. J. Thermophys..

[47] Silva W.C., Rocha A.M., Castro M.P.P., Sthel M.S., Vargas H., David G.F., Perez V.H. (2014). Unconventional characterization of biodiesel from several sources by thermal lens spectroscopy to determine thermal diffusivity: Phenomenological correlation among their physicochemical and rheological properties. Fuel.

[48] Savi E.L., Herculano L.S., Lukasievicz G.V.B., Regatieri H.R., Torquato A.S., Malacarne L.C., Astrath N.G.C. (2018). Assessing thermal and optical properties of biodiesel by thermal lens spectrometry: Theoretical and experimental aspects. Fuel.

[49] Starobor A.V., Mironov E.A., Volkov M.R., Karimov D.N., Ivanov I.A., Lovchev A.V., Naumov A.K., Semashko V.V., Palashov O.V. (2020). Thermal lens investigation in EuF2.11, PrF3, and Na0.38Ho0.62F2.24 crystals for magnetooptical applications. Opt. Mater..

[50] Francis F., Anila E.I., Joseph S.A. (2020). Dependence of thermal diffusivity on nanoparticle shape deduced through thermal lens technique taking ZnO nanoparticles and nanorods as inclusions in homogeneous dye solution. Optik.

[51] Thomas L., John J., Kumar B.R., George N.A., Kurian A. (2015). Thermal Diffusivity of Gold Nanoparticle Reduced by Polyvinyl Alcohol Using Dual Beam Thermal Lens Technique. Mater. Today Proc..

[52] Luna-Sánchez J.L., Jiménez-Pérez J.L., Carbajal-Valdez R., Lopez-Gamboa G., Pérez-González M., Correa-Pacheco Z.N. (2019). Green synthesis of silver nanoparticles using Jalapeño Chili extract and thermal lens study of acrylic resin nanocomposites. Thermochim. Acta.

[53] Nideep T.K., Ramya M., Nampoori V.P.N., Kailasnath M. (2020). The size dependent thermal diffusivity of water soluble CdTe quantum dots using dual beam thermal lens spectroscopy. Phys. E Low-Dimens. Syst. Nanostructures.

[54] Martins V.M., Brasse G., Doualan J.L., Braud A., Camy P., Messias D.N., Catunda T., Moncorgé R. (2014). Thermal conductivity of Nd^3+^ and Yb^3+^ doped laser materials measured by using the thermal lens technique. Opt. Mater..

[55] Martins V.M., Messias D.N., Doualan J.L., Braud A., Camy P., Dantas N.O., Catunda T., Pilla V., Andrade A.A., Moncorgé R. (2015). Thermo-optical properties of Nd3+ doped phosphate glass determined by thermal lens and lifetime measurements. J. Lumin..

[56] Sampaio J.A., Catunda T., Gama S., Baesso M.L. (2001). Thermo-optical properties of OH-free erbium-doped low silica calcium aluminosilicate glasses measured by thermal lens technique. J. Non-Cryst. Solids.

[57] Anjos V., Andrade A.A., Bell M.J.V. (2008). Thermal lens investigation in amorphous SiN. Appl. Surf. Sci..

[58] Ramis-Ramos G. (1993). Analytical characteristics, applications and perspectives in thermal lens spectrometry. Anal. Chim. Acta.

[59] Cedeno E., Cabrera H., Delgadillo-Lopez A.E., Delgado-Vasallo O., Mansanares A.M., Calderon A., Marin E. (2017). High sensitivity thermal lens microscopy: Cr-VI trace detection in water. Talanta.

[60] Shokoufi N., Yoosefian J. (2016). Selective determination of Sm (III) in lanthanide mixtures by thermal lens microscopy. J. Ind. Eng. Chem..

[61] Franko M., Liu M., Boskin A., Delneri A., Proskurnin M.A. (2016). Fast Screening Techniques for Neurotoxigenic Substances and Other Toxicants and Pollutants Based on Thermal Lensing and Microfluidic Chips. Anal. Sci..

[62] Liu M., Malovrh S., Franko M. (2016). Microfluidic flow-injection thermal-lens microscopy for high-throughput and sensitive analysis of sub-μL samples. Anal. Methods.

[63] Solimini D. (1966). Accuracy and sensitivity of the thermal lens method for measuring absorption. Appl. Opt..

[64] Liu M., Franko M. (2014). Progress in thermal lens spectrometry and its applications in microscale analytical devices. Crit. Rev. Anal. Chem..

[65] Mohebbifar M.R. (2021). Study of the effect of temperature on thermophysical properties of ethyl myristate by dual-beam thermal lens technique. Optik.

[66] Cruz R.A., Filadelpho M.C., Castro M.P., Andrade A.A., Souza C.M., Catunda T. (2011). Very low optical absorptions and analyte concentrations in water measured by Optimized Thermal Lens Spectrometry. Talanta.

[67] Liu M., Novak U., Plazl I., Franko M. (2013). Optimization of a Thermal Lens Microscope for Detection in a Microfluidic Chip. Int. J. Thermophys..

[68] Hannachi R. (2021). Photothermal lens spectrometry: Experimental optimization and direct quantification of permanganate in water. Sens. Actuat. B.

[69] Nossir N., Dalil-Essakali L., Belafhal A. (2018). Analytical study of flat-topped beam characterization using the thermal lens method in sample liquids. Optik.

[70] Shen J., Lowe R.D., Snook R.D. (1992). A model for cw laser induced mode-mismatched dual-beam thermal lens spectrometry. Chem. Phys..

[71] Ivshukov D.A., Mikheev I.V., Volkov D.S., Korotkov A.S., Proskurnin M.A. (2018). Two-Laser Thermal Lens Spectrometry with Signal Back-Synchronization. J. Anal. Chem..

[72] De Araujo M.A., Silva R., de Lima E., Pereira D.P., de Oliveira P.C. (2009). Measurement of Gaussian laser beam radius using the knife-edge technique: Improvement on data analysis. Appl. Opt..

[73] Skinner D.R., Whitcher R.E. (1972). Measurement of the radius of a high-power laser beam near the focus of a lens. J. Phys. E Sci. Instrum..

[74] Khosrofian J.M., Garetz B.A. (1983). Measurement of a Gaussian laser beam diameter through the direct inversion of knife-edge data. Appl. Opt..

[75] Tishchenko K., Muratova M., Volkov D., Filichkina V., Nedosekin D., Zharov V., Proskurnin M. (2017). Multi-wavelength thermal-lens spectrometry for high-accuracy measurements of absorptivities and quantum yields of photodegradation of a hemoprotein–lipid complex. Arab. J. Chem..

[76] Proskurnin M.A., Slyadnev M.N., Tokeshi M., Kitamori T. (2003). Optimisation of thermal lens microscopic measurements in a microchip. Anal. Chim. Acta.

[77] Proskurnin M.A., Kuznetsova V.V. (2000). Optimisation of the optical scheme of a dual-beam thermal lens spectrometer using expert estimation. Anal. Chim. Acta.

[78] Dovichi N.J. (1990). Laser-based microchemical analysis. Rev. Sci. Instrum..

[79] Dovichi N.J., Harris J.M. (1981). Thermal lens calorimetry for flowing samples. Anal. Chem..

[80] Dovichi N.J., Harris J.M. (1981). Time-resolved thermal lens calorimetry. Anal. Chem..

[81] Proskurnin M.A., Chernysh V.V., Filichkina V.A. (2004). Some Metrological Aspects of the Optimization of Thermal-Lens Procedures. J. Anal. Chem..

[82] Lima S.M., Sampaio J.A., Catunda T., Bento A.C., Miranda L.C.M., Baesso M.L. (2000). Mode-mismatched thermal lens spectrometry for thermo-optical properties measurement in optical glasses: A review. J. Non-Cryst. Solids.

[83] Ventura M., Simionatto E., Andrade L.H.C., Simionatto E.L., Riva D., Lima S.M. (2013). The use of thermal lens spectroscopy to assess oil–biodiesel blends. Fuel.

[84] Savi E.L., Malacarne L.C., Baesso M.L., Pintro P.T.M., Croge C., Shen J., Astrath N.G.C. (2015). Investigation into photostability of soybean oils by thermal lens spectroscopy. Spectrochim. Acta A Mol. Biomol. Spectrosc..

[85] Lenart V.M., Astrath N.G.C., Turchiello R.F., Goya G.F., Gómez S.L. (2018). Thermal diffusivity of ferrofluids as a function of particle size determined using the mode-mismatched dual-beam thermal lens technique. J. Appl. Phys..

[86] Kumar P., Khan A., Goswami D. (2014). Importance of molecular heat convection in time resolved thermal lens study of highly absorbing samples. Chem. Phys..

[87] Malacarne L.C., Astrath N.G., Medina A.N., Herculano L.S., Baesso M.L., Pedreira P.R., Shen J., Wen Q., Michaelian K.H., Fairbridge C. (2011). Soret effect and photochemical reaction in liquids with laser-induced local heating. Opt. Express.

[88] Martín-Biosca Y., Medina-Hernández M.J., García-Alvarez-Coque M.C., Ramis-Ramos G. (1994). Effect of the nature of the solvent on the limit of detection in thermal lens spectrometry. Anal. Chim. Acta.

[89] Cabrera H., Goljat L., Korte D., Marin E., Franko M. (2020). A multi-thermal-lens approach to evaluation of multi-pass probe beam configuration in thermal lens spectrometry. Anal. Chim. Acta.

[90] Prakash A., Pathrose B.P., Nampoori V.P.N., Radhakrishnan P., Mujeeb A. (2019). Thermal diffusivity of neutral red dye using dual beam thermal lens technique: A comparison on the effects using nano pulsed laser ablated silver and gold nanoparticles. Phys. E Low-Dimens. Syst. Nanostruct..

[91] Proskurnin M.A., Usoltseva L.O., Volkov D.S., Nedosekin D.A., Korobov M.V., Zharov V.P. (2021). Photothermal and Heat-Transfer Properties of Aqueous Detonation Nanodiamonds by Photothermal Microscopy and Transient Spectroscopy. J. Phys. Chem. C.

[92] Sharma N., Sharma N., Srinivasan P., Kumar S., Balaguru Rayappan J.B., Kailasam K. (2018). Heptazine based organic framework as a chemiresistive sensor for ammonia detection at room temperature. J. Mater. Chem. A.

[93] Georges J. (1994). A single and simple mathematical expression of the signal for cw-laser thermal lens spectrometry. Talanta.

[94] Augustine A.K., Mathew S., Girijavallabhan C.P., Radhakrishnan P., Nampoori V.P.N., Kailasnath M. (2014). Size dependent variation of thermal diffusivity of CdSe nanoparticles based nanofluid using laser induced mode-matched thermal lens technique. J. Opt..

[95] Cheremisinoff N.P., Cheremisinoff N.P. (1998). Cake Filtration and Filter Media Filtration. Liquid Filtration.

[96] Cheremisinoff N.P., Cheremisinoff N.P. (1998). An Introduction to Liquid Filtration. Liquid Filtration.

[97] Li X., Ding X., Bian C., Wu S., Chen M., Wang W., Wang J., Cheng L. (2019). Hydrophobic drug adsorption loss to syringe filters from a perspective of drug delivery. J. Pharmacol. Toxicol. Methods.

[98] Raj V., Jithin J., Swapna M.S., Sankararaman S. (2019). Effect of duty cycle on photothermal phenomenon—A thermal lens study. Optik.

