# Supplementary material for: Accuracy of Measurements of Thermophysical Parameters by Dual-Beam Thermal-Lens Spectrometry
Source: Nanomaterials (Basel). 2023 Jan 20;13(3):430. doi: 10.3390/nano13030430 (PMC9920435; doi:10.3390/nano13030430)
Supplement: Supplementary file 1 [file nanomaterials-13-00430-s001.zip › nanomaterials-2159924-supplementary.pdf]

nanomaterials

Supplementary Materials

for

Accuracy of Measurements of Thermophysical Parameters by Dual-Beam Thermal-Lens Spectrometry

Vladislav R. Khabibullin [1], Mladen Franko [2], and Mikhail A. Proskurnin [1,*]

[1] Analytical Chemistry Division, Chemistry Department, M.V. Lomonosov Moscow State University, d. 1, str. 3, Lenin Hills, GSP-1 V-234, Moscow 119991, Russia; vladhab1995@gmail.com
[2] Laboratory for Environmental and Life Sciences, University of Nova Gorica, Vipavska 13, Rožna Dolina, 5000 Nova Gorica, Slovenia; mladen.franko@ung.si
* Correspondence: proskurnin@gmail.com; Tel.: +7-(495)-939-46-48

Table. S1. Nomenclature of thermal-lens measurements.

Symbol	Transcript
A	Absorbance
α	Linear absorption coefficient
c	Concentration, mol/L
d_{av}	Average distance between molecules, nm
D	Thermal diffusivity, mm^2/sec
$\tilde{D}$	Effective thermal diffusivity, mm^2/sec
$\mathrm{d}n/\mathrm{d}T$	Temperature coefficient of the refractive index
$I(t)$	Intensity of the probe beam in time, mV
$\tilde{I}(t)$	Normalized (the range of 0–1) intensity of the probe beam in time
$I(0)$	Intensity of the probe beam at time t = 0, mV
$I(\infty)$	Intensity of the probe beam when the stationary (steady) state, mV
Φ	Phase shift
f_e	Focal length of the excitation laser lens, mm
f_p	Focal length of the probe laser lens, mm
k	Thermal conductivity, W/(m·K)
l	Cell path length, cm
m	Ratio of the radii of the probe beam to the excitation beam (mode-mismatch parameter)
P	Excitation laser power, W
t	Time
T	Temperature, K
t_c	Characteristic time, ms
$t_{c,theor}$	Theoretical value (true value) of the characteristic time, ms
$t_{c,exp}$	Apparent characteristic (experimental value) time, ms
$\tilde{t_c}$	Effective characteristic time, ms
ν	Modulator (Shutter) frequency, Hz
V	Distance parameter
z_1	Distance between the probe beam waist and the sample
z_2	Distance between the sample and the detector
z_{ce}	Confocal distance for probe beam (Rayleigh length for probe beam), mm
z_{cp}	Confocal distance for excitation beam (Rayleigh length for excitation beam), mm
λ_p	Wavelengths of the probe lasers, nm
λ_e	Wavelengths of the excitation lasers, nm
θ	Thermooptical signal
ω_{e0}	Radius of the waist of the excitation beam, µm
ω'_{e0}	Incorrect radius of the excitation beam, µm
ω_{p0}	Radius of the waist of the probe beam, µm
ω_{p1}	Radius of the probe beam in the sample, µm
Δ	Absolute error, %

Table. S2. Examples of geometric parameters of spectrometers for dual-beam TLS in the far field, where measurements are carried out according to the Shen-Snook model.

Purpose of the work	ω_{e0}, μm	ω_{p1}, μm	m	V	z_2, m	Ref.
Shen–Snook model	46.3	236	26.0	1.73	5	[1]
	80	190	5.6	1.73	6	
Measurement of thermal diffusivity of nanofluids	20.4	338.3	47.93	6.01	3.82	[2]
Biodiesel Analysis	30.5	212	48	1.79	-	[3]
Investigation of thermooptical properties of glasses	37.2	144.5	15.089	2.092	-	[4]
Measurement of the thermal diffusivity of aqueous dispersions of NPs	38	404	36.9	9.35	4.04	[5]
Investigation of water–ethanol systems	40	100	6.3		-	[6]
Measurement of thermal diffusivity of dispersions of nanoparticles	40	190	22.6	-	-	[7,8]
Investigation of the solvent effect on TL	41.0	65.0	2.51	-	2.5	[9]
Investigation of water-organic binary systems	41	100	6.3	-	-	[10]
Investigation of the thermal and optical properties of glasses	42	104	6.1	-	-	[11,12]
Investigate the thermooptical properties of glasses	43	195	20.6	1.81	2	[13]
Check the validity of the theoretical predictions	44.5	88.9	4.0	-	-	[14]
Analysis of soybean oil, biodiesel, and soybean oil–biodiesel blends	45.9	-	-	1.81	4	[15]
Measurement of thermal diffusivity of nanocomposites from acrylic resin	49	181	13.691	1.33	-	[16]
Investigation of the quantum yield and thermal diffusivity of glasses	49.7	218.4	19.3	-	-	[17]
Determination of lead(II)	50.3	71.1	2	-	1.19	[18]
Investigation of thermooptical properties of glasses	55	167	9.3	3	-	[19]
Check the validity of the theoretical predictions	56.2	43.3	0.59	-	-	[14]
Investigation of aqueous fullerene dispersions	58.5	82.1	1.97	1.01	-	[20]
Determination of the chelate concentration adsorbed on a quartz surface	60	84.9	2.0	3.1	1.2	[21]
Investigation of the quantum yield and thermal diffusivity of glasses	62	88	2.0		-	[12]
Investigation of the quantum yield and thermal diffusivity of glasses	62.6	90.5	2.1		-	[12]
Analysis of biodiesel fuel	63.0	539.5	73.3	3.99	4.0	[22]
Investigation of the thermooptical properties of amorphous SiN	64	205.4	10.3	1.7	-	[23]
Determination of copper(I)	65	94.2	2.1	3.1	0.9	[24]
Investigation of thermooptical properties of glasses	66.5	206	9.6	4	-	[19]
Investigation of the photostability of soybean oil	68	366	29	6.5	4.5	[25]
Investigation of the thermal diffusivity of NPs	92.3	195	4.46	-	-	[26]
Monitoring of the ester production	100	240.8	5.8	1.2	-	[27]
Analysis of diesel/biodiesel blends	119	429	13	3.88	-	[28]

Analysis of biodiesel fuel	188.0	675.0	12.9	14.2	4.0	[22]
Investigation of the quantum yield of doped glasses	-	-	30	19	-	[29]
This work	33.0	60.0	3.3	2.74	0.6	
	42.0	60.0	2.0	2.74	2.3-3.1	
	82.0	100	1.5	5.61		

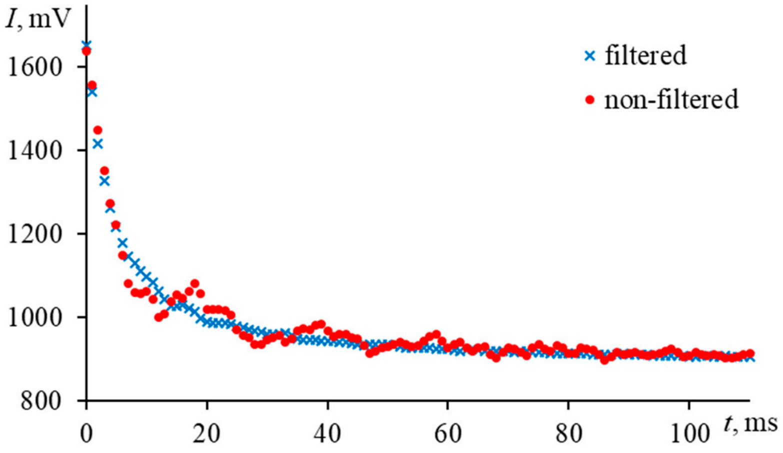

Figure S1. Transient curves for filtered and non-filtered aqueous solution of ferroin (2.5 μmol/L); narrow-focused configuration (ω_{e0} = 33 μm, Figure 1, Table 3).

References

1. Shen, J.; Lowe, R.D.; Snook, R.D. A model for cw laser induced mode-mismatched dual-beam thermal lens spectrometry. *Chemical Physics* **1992**, *165*, 385-396, doi:10.1016/0301-0104(92)87053-C.
2. Lopes, C.S.; Lenart, V.M.; Turchiello, R.F.; Gómez, S.L. Determination of the Thermal Diffusivity of Plasmonic Nanofluids Containing PVP-Coated Ag Nanoparticles Using Mode-Mismatched Dual-Beam Thermal Lens Technique. *Advances in Condensed Matter Physics* **2018**, *2018*, 1-6, doi:10.1155/2018/3052793.
3. Silva, W.C.; Rocha, A.M.; Castro, M.P.P.; Sthel, M.S.; Vargas, H.; David, G.F.; Perez, V.H. Unconventional characterization of biodiesel from several sources by thermal lens spectroscopy to determine thermal diffusivity: Phenomenological correlation among their physicochemical and rheological properties. *Fuel* **2014**, *130*, 105-111, doi:10.1016/j.fuel.2014.04.025.
4. Almeida, A.S.; Rivera, G.; Sousa, C.A.; Santos, F.E.P.; Souza, D.N. Thermal lens spectroscopy dosimetry at high doses using a commercial transparent glass. *Radiation Measurements* **2019**, *124*, 85-90, doi:10.1016/j.radmeas.2019.03.013.
5. Lenart, V.M.; Astrath, N.G.C.; Turchiello, R.F.; Goya, G.F.; Gómez, S.L. Thermal diffusivity of ferrofluids as a function of particle size determined using the mode-mismatched dual-beam thermal lens technique. *J. Appl. Phys.* **2018**, *123*, doi:10.1063/1.5017025.
6. Arnaud, N.; Georges, J. Investigation of the thermal lens effect in water–ethanol mixtures: composition dependence of the refractive index gradient, the enhancement factor and the Soret effect. *Spectrochimica Acta Part A* **2001**, *57*, 1295-1301, doi:10.1016/S1386-1425(00)00465-0.
7. Gutierrez Fuentes, R.; Pescador Rojas, J.A.; Jiménez-Pérez, J.L.; Sanchez Ramirez, J.F.; Cruz-Orea, A.; Mendoza-Alvarez, J.G. Study of thermal diffusivity of nanofluids with bimetallic nanoparticles with Au(core)/Ag(shell) structure. *Appl. Surf. Sci.* **2008**, *255*, 781-783, doi:10.1016/j.apsusc.2008.07.023.
8. Ramírez, J.F.S.; Pérez, J.L.J.; Valdez, R.C.; Orea, A.C.; Fuentes, R.G.; Herrera-Pérez, J.L. Thermal Diffusivity Measurements in Fluids Containing Metallic Nanoparticles using Transient Thermal Lens. *Int. J. Thermophys.* **2006**, *27*, 1181-1188, doi:10.1007/s10765-006-0084-8.

9. Colcombe, S.M.; Lowe, R.D.; Snook, R.D. Thermal lens investigation of the temperature dependence of the refractive index of aqueous electrolyte solutions. *Analytica Chimica Acta* **1997**, *356*, 277-288, doi:10.1016/S0003-2670(97)00475-3.
10. Arnaud, N.; Georges, J. Cw-laser thermal lens spectrometry in binary mixtures of water and organic solvents: composition dependence of the steady-state and time-resolved signals. *Spectrochim. Acta. A Mol. Biomol. Spectrosc.* **2004**, *60*, 1817-1823, doi:10.1016/j.saa.2003.09.019.
11. Sampaio, J.A.; Catunda, T.; Gama, S.; Baesso, M.L. Thermo-optical properties of OH-free erbium-doped low silica calcium aluminosilicate glasses measured by thermal lens technique. *Journal of Non-Crystalline Solids* **2001**, *284*, 210-216, doi:10.1016/s0022-3093(01)00404-5.
12. Sampaio, J.A.; Gama, S.; Baesso, M.L.; Catunda, T. Fluorescence quantum efficiency of Er3+ in low silica calcium aluminate glasses determined by mode-mismatched thermal lens spectrometry. *Journal of Non-Crystalline Solids* **2005**, *351*, 1594-1602, doi:10.1016/j.jnoncrysol.2005.03.047.
13. Lima, S.M.; Steimacher, A.; Medina, A.N.; Baesso, M.L.; Petrovich, M.N.; Rutt, H.N.; Hewak, D.W. Thermo-optical properties measurements in chalcogenide glasses using thermal relaxation and thermal lens methods. *Journal of Non-Crystalline Solids* **2004**, *348*, 108-112, doi:10.1016/j.jnoncrysol.2004.08.134.
14. Brennetot, R.; Georges, J. Pulsed-laser mode-mismatched dual-beam thermal lens spectrometry: comparison of the time-dependent and maximum signals with theoretical predictions. *Spectrochimica Acta Part A: Molecular and Biomolecular Spectroscopy* **1998**, *54*, 111-122, doi:10.1016/s1386-1425(97)00201-1.
15. Ventura, M.; Simionatto, E.; Andrade, L.H.C.; Simionatto, E.L.; Riva, D.; Lima, S.M. The use of thermal lens spectroscopy to assess oil–biodiesel blends. *Fuel* **2013**, *103*, 506-511, doi:10.1016/j.fuel.2012.08.027.
16. Luna-Sánchez, J.L.; Jiménez-Pérez, J.L.; Carbajal-Valdez, R.; Lopez-Gamboa, G.; Pérez-González, M.; Correa-Pacheco, Z.N. Green synthesis of silver nanoparticles using Jalapeño Chili extract and thermal lens study of acrylic resin nanocomposites. *Thermochimica Acta* **2019**, *678*, doi:10.1016/j.tca.2019.178314.
17. Andrade, A.A.; Coutinho, M.F.; de Castro, M.P.P.; Vargas, H.; Rohling, J.H.; Novatski, A.; Astrath, N.G.C.; Pereira, J.R.D.; Bento, A.C.; Baesso, M.L.; et al. Luminescence quantum efficiency investigation of low silica calcium aluminosilicate glasses doped with Eu2O3 by thermal lens spectrometry. *Journal of Non-Crystalline Solids* **2006**, *352*, 3624-3627, doi:10.1016/j.jnoncrysol.2006.03.092.
18. Saavedra, R.; Soto, C.; Gómez, R.; Muñoz, A. Determination of lead(II) by thermal lens spectroscopy (TLS) using 2-(2′-thiazolylazo)-p-cresol (TAC) as chromophore reagent. *Microchemical Journal* **2013**, *110*, 308-313, doi:10.1016/j.microc.2013.04.019.
19. Martins, V.M.; Brasse, G.; Doualan, J.L.; Braud, A.; Camy, P.; Messias, D.N.; Catunda, T.; Moncorgé, R. Thermal conductivity of Nd3+ and Yb3+ doped laser materials measured by using the thermal lens technique. *Opt. Mater.* **2014**, *37*, 211-213, doi:10.1016/j.optmat.2014.05.027.
20. Mikheev, I.V.; Usoltseva, L.O.; Ivshukov, D.A.; Volkov, D.S.; Korobov, M.V.; Proskurnin, M.A. Approach to the Assessment of Size-Dependent Thermal Properties of Disperse Solutions: Time-Resolved Photothermal Lensing of Aqueous Pristine Fullerenes C60 and C70. *The Journal of Physical Chemistry C* **2016**, *120*, 28270-28287, doi:10.1021/acs.jpcc.6b08862.
21. Kononets, M.Y.; Proskurnin, M.A.; Bendrysheva, S.N.; Chernysh, V.V. Investigation of adsorption of nanogram quantities of iron(II) tris-(1,10-phenanthrolinate) on glasses and silica by thermal lens spectrometry. *Talanta* **2001**, *53*, 1221-1227, doi:10.1016/S0039-9140(00)00613-5.
22. Savi, E.L.; Herculano, L.S.; Lukasievicz, G.V.B.; Regatieri, H.R.; Torquato, A.S.; Malacarne, L.C.; Astrath, N.G.C. Assessing thermal and optical properties of biodiesel by thermal lens spectrometry: Theoretical and experimental aspects. *Fuel* **2018**, *217*, 404-408, doi:10.1016/j.fuel.2017.12.104.

23. Anjos, V.; Andrade, A.A.; Bell, M.J.V. Thermal lens investigation in amorphous SiN. *Appl. Surf. Sci.* **2008**, *255*, 698-700, doi:10.1016/j.apsusc.2008.07.011.
24. Proskurnin, M.A.; Chernysh, V.V.; Pakhomova, S.V.; Kononets, M.Y.; Sheshenev, A.A. Investigation of the reaction of copper(I) with 2,9-dimethyl-1,10-phenanthroline at trace level by thermal lensing. *Talanta* **2002**, *57*, 831-839, doi:10.1016/S0039-9140(02)00128-5.
25. Savi, E.L.; Malacarne, L.C.; Baesso, M.L.; Pintro, P.T.M.; Croge, C.; Shen, J.; Astrath, N.G.C. Investigation into photostability of soybean oils by thermal lens spectroscopy. *Spectrochim. Acta. A Mol. Biomol. Spectrosc.* **2015**, *145*, 125-129, doi:10.1016/j.saa.2015.02.106.
26. Shahriari, E.; Varnamkhasti, M.G.; Zamiri, R. Characterization of thermal diffusivity and optical properties of Ag nanoparticles. *Optik - International Journal for Light and Electron Optics* **2015**, *126*, 2104-2107, doi:10.1016/j.ijleo.2015.05.086.
27. Deus, W.B.; Ventura, M.; Silva, J.R.; Andrade, L.H.C.; Catunda, T.; Lima, S.M. Monitoring of the ester production by near-near infrared thermal lens spectroscopy. *Fuel* **2019**, *253*, 1090-1096, doi:10.1016/j.fuel.2019.05.097.
28. Ventura, M.; Deus, W.B.; Silva, J.R.; Andrade, L.H.C.; Catunda, T.; Lima, S.M. Determination of the biodiesel content in diesel/biodiesel blends by using the near-near-infrared thermal lens spectroscopy. *Fuel* **2018**, *212*, 309-314, doi:10.1016/j.fuel.2017.10.069.
29. Figueiredo, M.S.; Santos, F.A.; Yukimitu, K.; Moraes, J.C.S.; Silva, J.R.; Baesso, M.L.; Nunes, L.A.O.; Andrade, L.H.C.; Lima, S.M. Luminescence quantum efficiency at 1.5µm of Er3+-doped tellurite glass determined by thermal lens spectroscopy. *Opt. Mater.* **2013**, *35*, 2400-2404, doi:10.1016/j.optmat.2013.06.041.
